# 3D Atlas of the Pituitary Gland of the Model Fish Medaka (*Oryzias latipes*)

**DOI:** 10.3389/fendo.2021.719843

**Published:** 2021-08-23

**Authors:** Muhammad Rahmad Royan, Khadeeja Siddique, Gergely Csucs, Maja A. Puchades, Rasoul Nourizadeh-Lillabadi, Jan G. Bjaalie, Christiaan V. Henkel, Finn-Arne Weltzien, Romain Fontaine

**Affiliations:** ^1^Physiology Unit, Faculty of Veterinary Medicine, Norwegian University of Life Sciences, Ås, Norway; ^2^Institute of Basic Medical Sciences, University of Oslo, Oslo, Norway

**Keywords:** pituitary, atlas, teleost, hormone, multihormonal cells, medaka, single-cell transcriptomics

## Abstract

In vertebrates, the anterior pituitary plays a crucial role in regulating several essential physiological processes *via* the secretion of at least seven peptide hormones by different endocrine cell types. Comparative and comprehensive knowledge of the spatial distribution of those endocrine cell types is required to better understand their physiological functions. Using medaka as a model and several combinations of multi-color fluorescence *in situ* hybridization, we present the first 3D atlas revealing the gland-wide distribution of seven endocrine cell populations: lactotropes, thyrotropes, Lh and Fsh gonadotropes, somatotropes, and *pomca*-expressing cells (corticotropes and melanotropes) in the anterior pituitary of a teleost fish. By combining *in situ* hybridization and immunofluorescence techniques, we deciphered the location of corticotropes and melanotropes within the *pomca*-expressing cell population. The 3D localization approach reveals sexual dimorphism of *tshba*-, *pomca*-, and *lhb*-expressing cells in the adult medaka pituitary. Finally, we show the existence of bi-hormonal cells co-expressing *lhb*-*fshb*, *fshb*-*tshba* and *lhb*-*sl* using single-cell transcriptomics analysis and *in situ* hybridization. This study offers a solid basis for future comparative studies of the teleost pituitary and its functional plasticity.

## Highlights

- We offer the first 3D atlas of a teleost pituitary, which presents a valuable resource to the endocrinology and model fish community.
- The atlas reveals the 3D spatial distribution of the seven endocrine cell types and blood vessels in the juvenile/adult male and female pituitary.
- Gene expression for *tshba*, *pomca*, and *lhb*, as well as the volume of the cell population expressing these genes, displays obvious sexual dimorphism in the adult medaka pituitary.
- Multi-color *in situ* hybridization and single cell RNA-seq reveal the existence of bi-hormonal cells, co-expressing *lhb*-*fshb*, *fshb*-*tshba*, *lhb*-*sl*, and a few multi-hormonal cells.
- An online version of the atlas is available at https://www.nmbu.no/go/mpg-atlas.

## Introduction

In vertebrates, the pituitary is considered the *chef d’orchestre* of the endocrine system, regulating several essential biological and physiological functions throughout the life cycle. Located beneath the hypothalamus, it is divided into the anterior part (adenohypophysis) and posterior part (neurohypophysis). The former comprises several endocrine cell types which produce and release specific peptide hormones, controlling many important aspects of life, including growth, stress, metabolism, homeostasis, and reproduction (1, 2).

During embryogenesis, different cellular developmental trajectories specify several endocrine cell types in the adenohypophysis, characterized by the hormones they produce (3). In general, the vertebrate adenohypophysis consists of lactotropes (producing prolactin; Prl), corticotropes (adrenocorticotropic hormone; Acth), thyrotropes (thyrotropin; Tsh), gonadotropes (follicle-stimulating and luteinizing hormone; Fsh and Lh), somatotropes (growth hormone; Gh), and melanotropes (melanocyte-stimulating hormone; α-Msh), which have specific roles in regulating certain physiological functions (2, 4). Teleosts, in addition, have an endocrine cell type that is unique to these animals, i.e. somatolactotropes (somatolactin; Sl) (5). In contrast to mammals and birds, Fsh and Lh are mostly secreted by distinct endocrine cell types in teleosts (6), although both transcripts or hormones have sometimes been observed in the same cells in some species (7–10). Unlike mammals, teleost endocrine cells are arranged in discrete zones. Lactotropes and corticotropes are commonly located in the *rostral pars distalis* (RPD), thyrotropes, gonadotropes, and somatotropes in the *proximal pars distalis* (PPD), and melanotropes and somatolactoropes in the *pars intermedia* (PI) (5, 11).

Over the past five decades, endocrine cell type organization in the teleost pituitary has been documented in various species. Despite having approximately similar patterns, the pituitary endocrine cell maps exhibit differences in terms of variety of cell types that are reported. For instance, the localization of endocrine cell populations in dorado fish shows only four distinct types of endocrine cells across the adenohypophysis (12). By contrast, studies of other species described five [Japanese medaka (13)], six [fourspine sculpin (14); cardinal and bloodfin tetra (15)], seven [greater weever (16); white seabream (17); dimerus cichlid (18)], and eight [Atlantic halibut (19); Nile tilapia (20); saddle wrasse (21)] cell types.

Even though these previous studies have provided interesting information on the spatial organization of endocrine cell populations, they lack information due to the techniques available and used at the time. First, the use of mid- and para-sagittal sections of the pituitary to reconstruct organizational patterns of endocrine cells overlooks information on the lateral sides. Second, the single-labeling method and non-species specific antibodies typically used do not provide sufficient detail on arrangements among different endocrine cell populations, or on the possible existence of multi-hormonal cells as described in mammals (22–25). These features will be important to better understand their roles in fish physiology and endocrinology. Moreover, the distribution of the blood vessels within the pituitary, which play an essential role by transporting the released hormones, is poorly known. A better knowledge will help understand how endocrine cells arranged within a vascularized system that is thought to facilitate signaling within the pituitary (26). Also, since it has been shown that the pituitary is a plastic organ with changes occurring at cellular and population levels (27), it is essential to describe the cell composition, spatial organization, and vascularization of the pituitary in detail.

The Japanese medaka (*Oryzias latipes*) is a teleost model commonly used to investigate vertebrate and teleost physiology, genetics, and development, due to easy access to a wide range of genetic and molecular techniques (28, 29). We have recently used single-cell RNA sequencing to describe seven distinct endocrine cell types (expressing *prl*, *pomca*, *fshb*, *lhb*, *tshba*, *gh*, and *sl*) in the medaka pituitary (30). Here, we extend this study by describing differences in the spatial distribution of the seven endocrine cell populations, in juvenile and adult fish from both sexes. Using multi-color *in situ* hybridization techniques together with single-cell transcriptomics analysis, this study offers the first 3D atlas of teleost pituitary endocrine cell populations, allowing the characterization of differences in spatial distribution between sexes and stages, as well as demonstrating the existence of multi-hormonal cells.

## Materials and Methods

### Experimental Animals

Juvenile (2 months old) and adult (6 months old) wild type medaka (WT, d-rR strain) were reared at 28°C in a re-circulating water system (pH 7.5; 800 µS) with 14 hours light and 10 hours dark. Fish were fed with artemia and dry food three times daily. Sex determination was based on secondary sexual characteristics (31). Experiments were conducted in accordance with recommendations on experimental animal welfare at the Norwegian University of Life Sciences.

### Quantitative Polymerase Chain Reaction

RNA extraction from pituitaries (n = 7) was performed as previously described in (32). Fish were euthanized by immersion in ice water and pituitaries were collected and stored at -80°C in 300 µl of TRIzol (Invitrogen, Carlsbad, USA) with 6 zirconium oxide beads (Bertin Technologies, Versailles, France; diameter 1.4 μm). Later, tissues were homogenized and mixed with 120 µl chloroform. After centrifugation, the supernatant was mixed with isopropanol, and the RNA pellet was rinsed with 75% cold ethanol before resuspended with 14 µl of nuclease free water. Due to the size of the tissue, 3 juvenile pituitaries were pooled per replicate. A total of 33 ng of RNA was used to synthesize cDNA using SuperScript III Reverse Transcriptase (Invitrogen, Carlsbad, CA, USA) and random hexamer primers (Thermofisher scientific). 5× diluted cDNA samples were analyzed in duplicate, using 3 µl of the cDNA and 5 µM each of forward and reverse primer in a total volume of 10 µl (**Table 1**). PCR cycle parameters were: 10 min pre-incubation at 95°C, followed by 42 cycles of 95°C for 10 s, 60°C for 10 s and 72°C for 6 s, followed by melting curve analysis to assess PCR specificity. The mRNA level was normalized using *rpl7* as the reference gene as no significant difference of expression was found between groups.

**Table 1:** Primer sequences used for the mRNA level analysis in the medaka pituitary.

Gene Name	Sequence (5’ - 3‘)	Ensembl Gene Name	Accession Number (NCBI/Ensembl)	Amplicon size (bp)	Efficiency	Reference
*rpl7*	F: TGCTTTGGTGGAGAAAGCTC	*rpl7*	NM_001104870	98	2.03	(32)
	R: TGGCAGGCTTGAAGTTCTTT		ENSORLG00000007967			
*prl*	F: TCAGATGGGAACCAGAGGAC	*prl1*	XM_004071867.4	85	1.987	This study
	R: GATGTCCACGGCTTTACACA		ENSORLG00000016928			
*tshba*	F: ATGTGGAGAAGCCAGAATGC	*tshba*	XM_004068796.4	88	2	This study
	R: CTCATGTTGCTGTCCCTTGA		ENSORLG00000029251			
*lhb*	F: CCACTGCCTTACCAAGGACC	*lhb*	NM_001137653.2	100	2	(33)
	R: AGGAAGCTCAAATGTCTTGTAG		ENSORLG00000003553			
*fshb*	F: GACGGTGCTACCATGAGGAT	*fshb*	NM_001309017.1	73	2.03	(32)
	R: TCCCCACTGCAGATCTTTTC		ENSORLG00000029237			
*gh*	F: TCGCTCTTTGTCTGGGAGTT	*gh1*	XM_004084500.3	102	1.94	This study
	R: ACATTCTGATTGGCCCTGAT		ENSORLG00000019556			
*pomca*	F: GTGGTGGTTGTCGGTGGG	*pomca*	XM_004066456.3	122	1.956	This study
	R: GTGAGGTCAGAGCGGCAG		ENSORLG00000025908			
*sl*	F: CACCAAAGCATTACCCATCC	*smtla*	NM_001104790.1	87	1.965	This study
	R: ACCAGCATCAGCACAGAATG		ENSORLG00000013460			

### Multi-Color Fluorescence *In Situ* Hybridization

#### Tissue Preparation

Fish were euthanized by immersion in ice water. Each brain and pituitary complex was fixed overnight at 4°C in 4% paraformaldehyde (PFA, Electron Microscopy Sciences, Hatfield, Pennsylvania) diluted with phosphate buffered saline with Tween (PBST: PBS, 0.1%; Tween-20), approximately 50× the tissue volume. Tissue was then dehydrated in a series of increasing ethanol concentrations (25%, 50%, 75%, 96%), followed by storage in 100% methanol at -20°C until use.

#### Cloning and RNA Probe Synthesis

DNA sequences for the probes were obtained from NCBI as listed in **Table 2**. Sequences were selected according to the high expression in the pituitary for those having more than one paralog in the medaka genome (*tshb* and *pomc*). PCR primers for the amplification of the probe genes were designed from transcribed sequences (mRNA) for each gene using Primer3 (https://primer3.ut.ee/). Following RNA extraction and cDNA synthesis as described above, cDNA was used to amplify the sequence of interest by PCR using Taq DNA polymerase (Thermo Fisher Scientific) with a 3-min denaturation step at 94°C, followed by 35 cycles at 94°C for 15s, 50°C for 15s, and 72°C for 60s, and finally 1 cycle of 72°C for 5 mins. The amplified PCR products were isolated using a gel extraction kit (Qiagen) and cloned into the pGEM-T Easy vector (Promega) following manufacturer instructions and verified by sequencing. PCR products from the verified plasmids were used as template to synthetize sense and anti-sense complementary RNA probes using *in vitro* transcription with T7 or SP6 RNA polymerase (Promega, Madison, Wisconsin). RNA probes were tagged with dinitrophenol-11-UTP (DNP, Perkin Elmer, Waltham, Massachusetts), fluorescein-12-UTP (FITC, Roche Diagnostics), or digoxigenin-11-UTP (DIG, Roche Diagnostics). Finally, the probes were purified using the Nucleospin RNA clean-up kit (Macherey-Nagel, Hoerdt, France) and the concentration was measured using the Epoch Spectrophotometer System (BioTek, Winooski, VT, USA).

**Table 2:** Primer sequences used to make the *in situ* hybridization (ISH) probes of seven endocrine cell types in the medaka pituitary.

Gene	Sequence (5’ - 3’)	Ensmbl gene name	Accession Number (NCBI/Ensembl)	PCR product size (bp)	Reference
*lhb*	F: CACAGCCTGCAGATACATGAG	*lhb*	NM_001137653.2	318	(33)
	R: AGGAAGCTCAAATGTCTTGTAG		ENSORLG00000003553		
*fshb*	F: GAGGAAGCAACACTTTCAGC	*fshb*	NM_001309017.1	500	(34)
	R: GCACAGTTTCTTTATTTCAGTGC		ENSORLG00000029237		
*pomca*	F: ATGTATACCGTTTGGTTGCT	*pomca*	XM_004066456.3	515	This study
	R: AAATGCTTCATCTTGTAGGAG		ENSORLG00000025908		
*sl*	F: CCCATCTTTTCACTGTAAGT	*smtla*	NM_001104790.1	506	This study
	R: ATACTGGAAGGCACCTTGTT		ENSORLG00000013460		
*prl*	F: GAAAGACCGAGGAGGAACTG	*prl1*	XM_004071867.4	381	This study
	R: TTGCAGAGTTGGACAGGACC		ENSORLG00000016928		
*gh*	F: TCTCTGCAGACTGAGGAACA	*gh1*	XM_004084500.3	501	This study
	R: AGCCACAGTCAGGTAGGTCT		ENSORLG00000019556		
*tshba*	F: ACAGGCTAAACTCAAGTTAA	*tshba*	XM_004068796.4	473	This study
	R: AGGATCATATAGGTGCTCTG		ENSORLG00000029251		

#### Hybridization

Multi-color FISH was performed as previously described in (35) with minor modifications. Tissues were serially rehydrated, and the pituitary was detached from the brain. Afterwards, whole pituitaries were hybridized with the probes (0.11 – 3.17 ng/µl) for 18 hours at 55°C, and incubated with different combinations of anti-DNP- (Perkin Elmer), anti-FITC-, and anti-DIG-conjugated antibodies (Roche Diagnostics), followed by TAMRA- (Thermofisher), Cy5- (Perkin Elmer) and FITC-conjugated tyramides (Sigma). The nuclei were stained with DAPI (1:1000, 4’, 6-diamidino-2-phenylindole dihydrochloride; Sigma). The absence of labeling when using sense probes was used to confirm the specificity of the anti-sense probes. Whole pituitaries were mounted using Vectashield H-1000 Mounting Medium (Vector, Eurobio/Abcys) between microscope slides and cover slips (Menzel Glässer, VWR) with spacers (Reinforcement rings, Herma) in between for the juveniles, and between two cover slips with spacers for adults.

### Combined FISH and Immunofluorescence

To distinguish the localization of adrenocorticotropic-releasing hormone (Acth) and alpha-melanocyte stimulating hormone (α-Msh) cells within *pomca*-expressing cells separately, IF was performed using the antibodies shown in **Table 3**. After FISH for *pomca* labelled with FITC-conjugated tyramide, the pituitaries were embedded in 3% agarose (H_2_O) and para-sagittally sectioned with 60 µm thickness using a vibratome (Leica). From a single pituitary, odd and even ordered slices were processed to detect Acth and α-Msh IF, respectively. Tissue slices were incubated for 10 minutes at room temperature (RT) in permeabilizing buffer (0.3% Triton in PBST) with agitation, before incubation for 1 hour at RT in blocking solution (Acth: 3% normal goat serum (NGS); 0.3% Triton; 1% dimethylsulfoxide (DMSO) in PBST; α-Msh: 3% NGS; 5% Triton; 7% DMSO in PBST). Sections were then incubated at 4°C overnight with primary antibodies or without (control) in blocking solution, followed by 4 hours at RT with secondary antibodies in blocking solution with extensive PBST washes in between. Nuclei were stained with DAPI (1/1000). Antibody dilution factors are provided in **Table 3**.

**Table 3:** Primary and secondary antibodies used for immunofluorescence (IF) to distinguish Acth and α-Msh cells within *pomca*-expressing cells in the medaka pituitary.

Antibody	Dilution	Source	Reference
Rabbit anti-human ACTH antibody	1:1000	abcam (ab74976)	(36)
Rabbit anti-human α-MSH antibody	1:3000	abcam (ab123811)	(37)
Goat anti-rabbit antibody (Alexa-555)	1:500	Invitrogen (A21429)	(34)

### Blood Vessel Staining

Blood vessels were stained by cardiac perfusion as previously described (38). The fish were anesthetized with 0.04% Tricaine (pH 7), and the anterior abdomen was cut to allow access to the heart. Afterwards, 0.05% of DiI (1,1’-Dioctadecyl-3,3,3’,3’-Tetramethylindocarbocyanine Perchlorate; Invitrogen) solution diluted in 4% PFA (in PBS) was administered to the *bulbus arteriosus* through the ventricle using a glass needle. The pituitary was dissected and fixed in 4% PFA (in PBS) for 2 hours in the dark, then washed 2 times with PBS and mounted as described above.

### Image Processing and Analysis

Fluorescent images were obtained using an LSM710 Confocal Microscope (Zeiss) with 25× (for adult pituitary) and 40× (for juvenile pituitary) objectives. Lasers with wavelength of 405 (DAPI), 555 (TAMRA; Alexa-555), 633 (Cy5) and 488 (FITC; Alexa-488) nm were used. Channels were acquired sequentially to prevent cross-signaling of fluorophores. Due to the size of the adult pituitaries, the image acquisition was done from the dorsal and ventral sides of the pituitary with some overlaps in the middle. In conjunction with the microscope, ZEN software (v2009, Zeiss) was used to process the images, and ImageJ (1.52p; http://rsbweb.nih.gov/ij/) was used for processing z-projections from confocal image stacks. The dorsal and ventral stacks of adult pituitaries were aligned using HWada (https://signaling.riken.jp/en/en-tools/imagej/635/) and StackReg plugins (http://bigwww.epfl.ch/thevenaz/stackreg/), before presenting them in orthogonal views.

### 3D Volume Measurement of Cell Populations

Cell volumes from the pituitary image stacks (n = 4-8 per group) were measured using ImageJ. Briefly, the channels of the image stack were split, and the threshold was adjusted to select only the objects of interest and to exclude background. The Otsu threshold method was used to separate foreground objects from background. To calculate the object volume, the area of the slice was multiplied by the depth of the slice. The absolute volume of each population was calculated by summing the population volume of each slice. Then, the relative volume of each population as a percent of the total pituitary volume (determined by DAPI staining) was calculated.

### 3D Atlasing

While juvenile pituitaries were imaged as one block, adult pituitaries were imaged from the ventral and dorsal side with confocal imaging as described above. The two sides of the adult pituitaries were then merged using landmarks visible with the DAPI staining. Finally, eight pituitaries labeled for different markers were aligned to the same coordinate system also using manually selected landmarks. These data were used for the creation of four 3D atlases of the pituitary gland, using the principle approaches previously outlined (39).

The creation of the 3D atlases involved several steps. Merging and alignment was done using LandmarkReg (https://github.com/Tevemadar/LandmarkReg, with accompanying utilities https://github.com/Tevemadar/LandmarkReg-utils). Image stacks were saved in NIfTI format (https://imagej.nih.gov/ij/plugins/nifti.html) and converted with the “NIfTI2TopCubes” utility before the matching anatomical positions (“landmarks”) were manually identified in volume-pairs. Both the signal from endocrine cells and the DAPI background were inspected, and four or more landmarks were recorded for each volume-pair. In case of ventral-dorsal half images (adult samples), a custom utility “PituBuild” was used for merging the two halves, based on partial overlap. Finally, each set of complete pituitary volumes was aligned to a common anatomical space using the “Match” utility. The resulting NIfTI volumes were then converted to TIFF stacks for viewing and analysis.

To enable 3D viewing of the pituitary atlases, data were prepared for the MeshView tool (RRID: SCR_017222, https://www.nitrc.org/projects/meshview/) developed for 3D brain atlas viewing (40). Volumes were first binarized using the “BinX” utility with a threshold value of 50. MeshGen (https://www.nitrc.org/projects/meshgen/) was then used to generate surface meshes in standard STL format (http://paulbourke.net/dataformats/stl/) before convertion using PackSTL (https://github.com/Tevemadar/MeshView-PackSTL) to allow viewing in MeshView.

### Single-Cell Transcriptomics Analysis (scRNA-Seq)

We used processed scRNA-seq dataset from 23 male and 24 female adult medaka pituitaries where doublet cells were removed (30). Briefly, 2644 and 3921 cells remained after quality control for the female and male pituitary, respectively. Then, each cell was awarded a doublet likelihood score in the doublet removal analysis. We removed 2-3% of the cells with the highest doublet score from the original dataset according to 10x Genomics stochastic loading data, ending with 2592 female and 3804 male cells. We then filtered out red blood cells to avoid noise, and we applied a cut-off to differentiate between cells with high and low expression for specific genes to distinguish expression from background (**Supplementary Figure 1**). This dataset was further used to generate the pair-wise scatterplots using the R package ggplot2 (version 2_3.3.2), to investigate cells expressing more than one hormone-encoding gene. We identified 191 and 229 multiple hormone-producing cells in female and male pituitaries, respectively. To identify whether these cells express more than two hormone-encoding genes, we generated clustered heatmaps using pheatmap (version 1.0.12) to visualize the expression levels of all hormone-producing genes in each cell.

### Statistical Analysis

Levene’s test was performed to analyze the homogeneity of variance while the normality was tested using the Shapiro-Wilk Normality test. Differences in mRNA levels and in absolute and relative cell population volumes were evaluated using One-way ANOVA followed by Tukey *post hoc* test. The data are shown as mean + SEM (Standard Error of Mean) unless otherwise stated in the figure legend. *p* < 0.05 was used as a threshold for statistical significance.

### Data Availability

**Figure 1:**
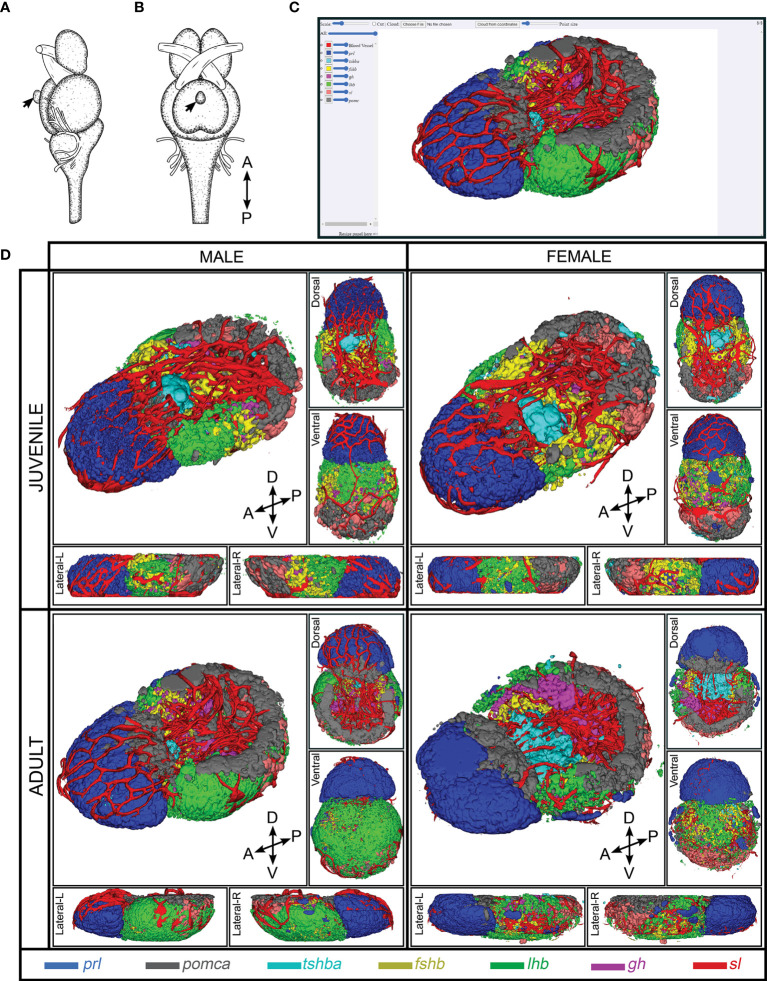
3D reconstruction of medaka anterior pituitary containing seven endocrine cell populations and blood vessel. Illustration of brain and pituitary (marked by black arrow) of medaka from lateral view **(A)** and ventral view **(B)** [Figure is adopted and modified with permission from (41)]. The navigation platform of 3D spatial distribution of endocrine cell populations that are now available online **(C)**. Snapshots of 3D reconstruction of endocrine cell populations from juvenile and adult medaka male and female **(D)**. Snapshots of 3D representations of the pituitary atlas captured from different perspectives: lateral (L, left; R, right), dorsal, and ventral. Four direction arrows display the direction of the pituitary (A, anterior; P, posterior; D, dorsal; V, ventral). The legend shows the color code for each cell type.

All image files are available in a data repository (https://doi.org/10.18710/NOGJQ2). The 3D atlases (**Figure 1**) can be found on a webpage containing explanatory videos and other types of data completing the online pituitary atlas (https://www.nmbu.no/go/mpg-atlas), allowing easy access and navigation through the data.

## Results

### 3D Atlases of the Medaka Pituitary

Using several combinations of multi-color FISH, we combined the labeling to form four 3D pituitary atlases now available online http://arken.nmbu.no/medaka-pituitary-atlas/JF/; http://arken.nmbu.no/medaka-pituitary-atlas/JM/; http://arken.nmbu.no/medaka-pituitary-atlas/AF/; http://arken.nmbu.no/medaka-pituitary-atlas/AM/), allowing us to precisely localize the seven endocrine cell types (*prl*, *pomca*, *tshba*, *fshb*, *lhb*, *gh* and *sl*) and blood vessels in the adenohypophysis of medaka.

#### *prl*-Expressing Cells (Lactotropes)

In both adults and juveniles (**Supplementary Figure 2**), *prl*-expressing cells make up almost the entirety of the RPD from the dorsal to the ventral side of the pituitary, without any obvious difference in distribution between males and females. They border on and intermingle with a *pomca*-expressing cell population (**Supplementary Figure 9**). In some fish, a few *prl*-expressing cells are also localized peripherally in the dorsal area of PPD (data not shown).

#### *pomca*-Expressing Cells (Corticotropes and Melanotropes)

*pomca*-expressing cells are observed in two distinct regions. One population is localized in the dorsal part of RPD, where it is mostly clustered centrally if observed from the transverse perspective, and the second is detected in the PI area (**Supplementary Figure 3**). While the first one is adjacent to and mixing with *prl*-expressing cells, in close proximity to *tshba*-expressing cells (**Supplementary Figure 9**), the second population intermingles with *sl*-expressing cells (**Supplementary Figure 10**).

#### *tshba*-Expressing Cells (Thyrotropes)

*tshba*-expressing cells are localized in the dorsal side of anterior PPD towards the *pars nervosa* (PN; analogous to neurohypophysis in the anterior part of the pituitary), next to the *prl*- and *pomca*-expressing cells (**Supplementary Figure 4** and **Supplementary Figure 9**). From the transverse perspective, *tshba*-expressing cells are mostly concentrated centrally in the PPD (**Supplementary Figure 4**) where they border and mix with *fshb*-expressing cells (**Supplementary Figure 11**).

#### *fshb*-Expressing Cells (Gonadotropes)

*fshb*-expressing cells are detected from the anterior to middle part of the PPD, distributed in both lateral sides of the pituitary from the transverse perspective (**Supplementary Figure 5**). These cells cover the PN from a frontal perspective (**Supplementary Figure 5**). They border and mix with *tshba*-expressing cells in the dorsal (**Supplementary Figure 11**), *lhb*-expressing cells in the ventral (**Supplementary Figure 12**) and *gh*-expressing cells in the posterior part of the PPD (**Supplementary Figure 13**).

#### *lhb*-Expressing Cells (Gonadotropes)

In both juveniles and adults, *lhb*-expressing cells are commonly distributed in the peripheral area of the PPD, covering almost the entire ventral side of the pituitary (**Supplementary Figure 6**). In adults, *lhb*-expressing cells are also localized in the proximity of peripheral area of the PI (**Supplementary Figure 6**). These cells border and mix with *fshb*-expressing cells in the PPD (**Supplementary Figure 12**), and with *pomca*- and *sl*-expressing cells in the PI of adult pituitary (**Supplementary Figure 10**).

#### *gh*-Expressing Cells (Somatotropes)

*gh*-expressing cells are localized on the dorsal side of the PPD towards the PN area (**Supplementary Figure 7**). They are distributed in both lateral sides of the pituitary, mixing with *fshb*-expressing cells (**Supplementary Figure 13**), but extend posteriorly, encompassing and bordering with the PN (**Supplementary Figure 7**).

#### *sl*-Expressing Cells (Somatolactotropes)

*sl*-expressing cells are intermingled within *pomca*-expressing cells located in the PI (**Supplementary Figure 8** and **Supplementary Figure 10**). In the adult pituitary, these cells border and mix with *lhb*-expressing cells in the PI (**Supplementary Figure 10**).

#### Distinction of Acth and α-Msh Cell Populations

**Figure 2:**
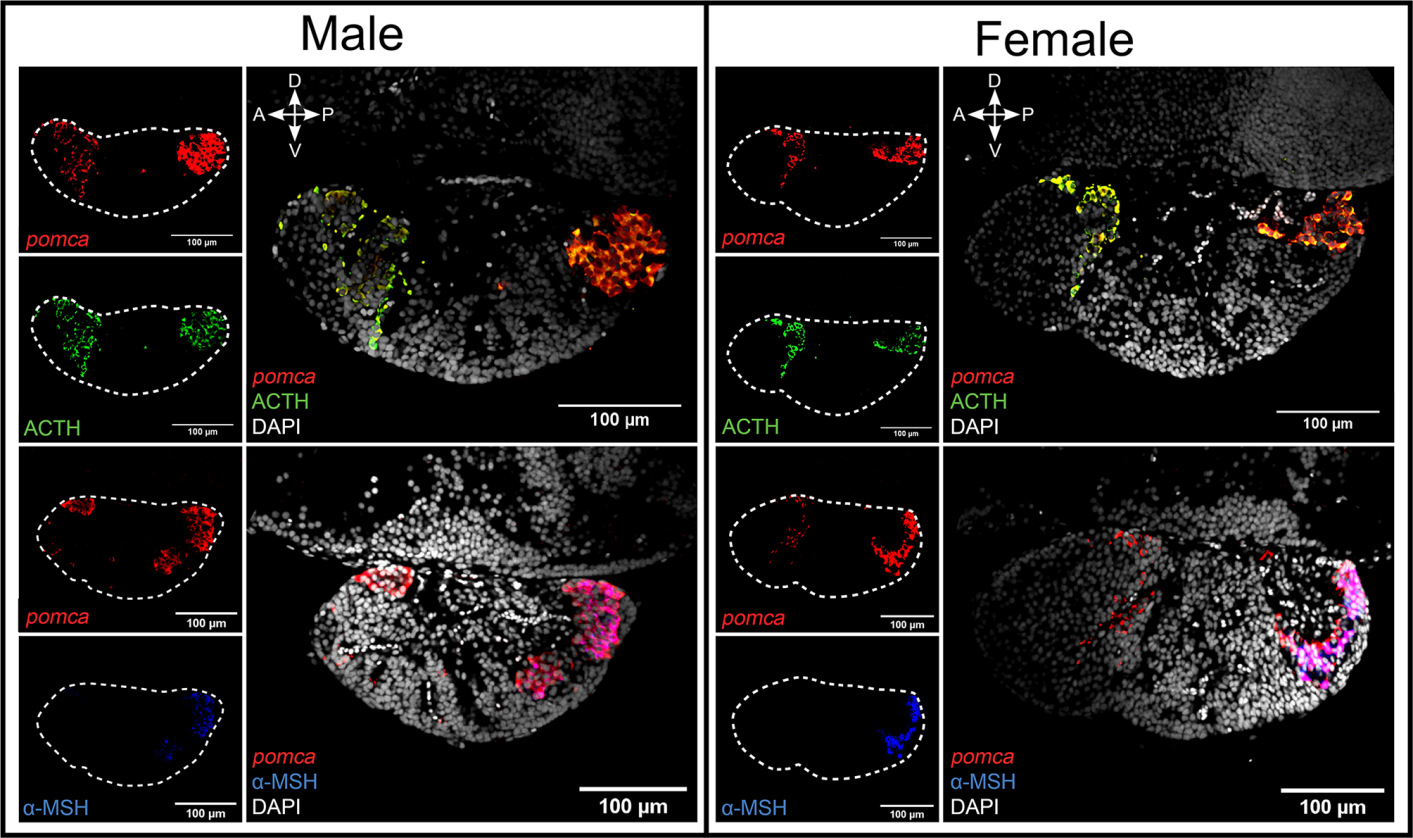
The combination of FISH for *pomca* and IF for Acth or α-Msh allows the distinction of two clear *pomca* expressing cell populations. The distinction of Acth (green) and α-Msh (blue) producing cells from *pomca*-labelled (red) in the pituitary from adult male and female medaka. The dashed lines represent the pituitaries shown in the right panel. Four direction arrows display the direction of the pituitary (A, anterior; P, posterior; D, dorsal; V, ventral).

The combination of FISH for *pomca* with IF for Acth or α-Msh shows that that Acth cells overlap the entire *pomca* signal, while melanotropes overlap *pomca* signals in the PI, both in adults (**Figure 2**) and in juvenile pituitaries (**Supplementary Figure 14**).

#### Blood Vessels

**Figure 3:**
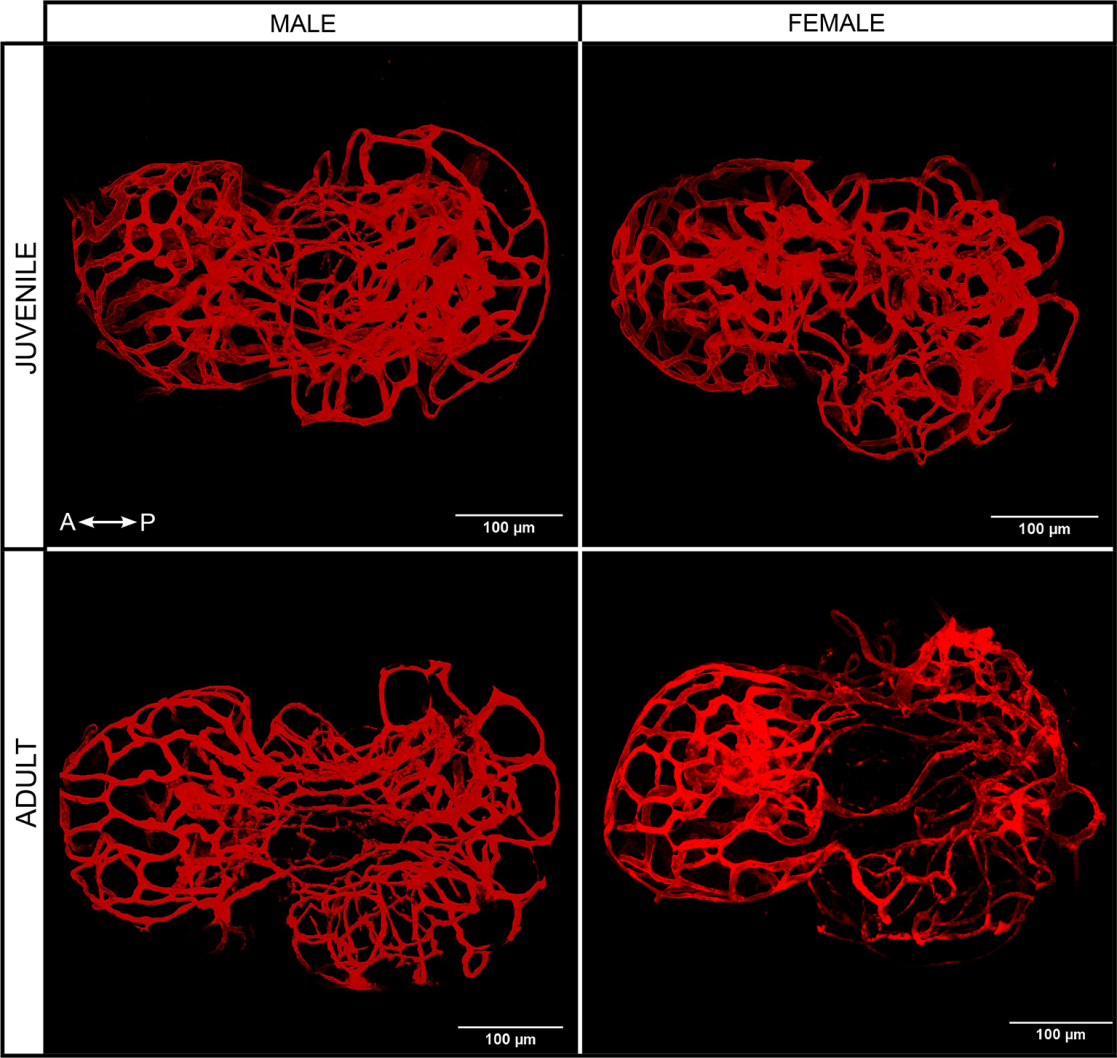
3D projection of blood vessels from juvenile and adult male and female medaka pituitaries from the dorsal side. Left right arrow symbol shows the direction of the pituitary (A, anterior; P, posterior).

3D reconstruction shows that blood vessels encompass the entire adenohypophysis, without any obvious differences between sexes and stages (**Figure 3**).

### Sex and Stage Differences

**Figure 4:**
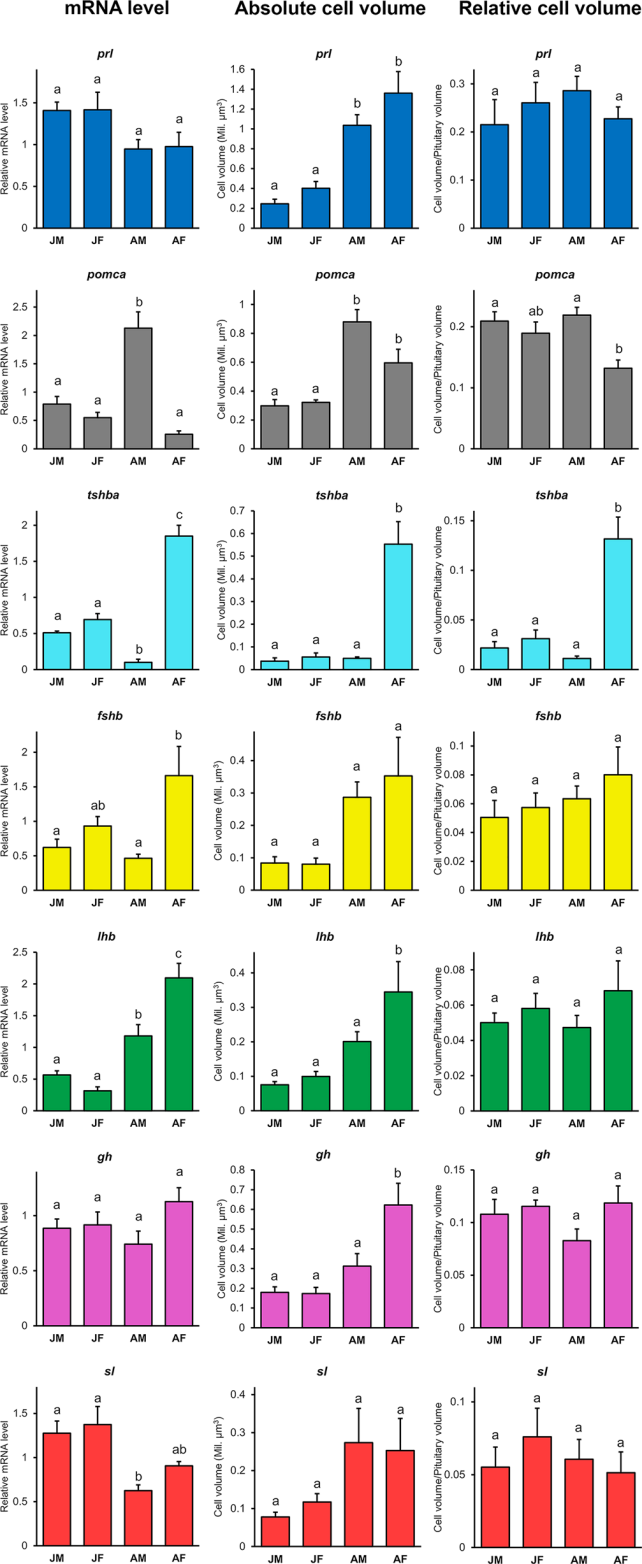
Relative mRNA levels for seven hormone-encoding genes (*prl*, *pomca, tshba*, *fshb*, *lhb*, *gh*, and *sl*), absolute cell population volumes and relative cell volumes in juvenile and adult medaka males and females (juvenile male, JM; juvenile female, JF; adult male, AM; adult female, AF). Graphs are provided as mean + SEM (n = 4 - 8). Different letters display statistical differences (*p* < 0.05) between groups as evaluated by One-way ANOVA followed by Tukey *post hoc* test.

For each pituitary endocrine cell population, we analyzed the mRNA levels for the hormone-encoding genes, as well as the absolute and relative volumes of each population (**Figure 4**).

Although no stage difference is observed in *prl* and *gh* mRNA levels, the *prl*-expressing cell volume is significantly larger in adults than in juveniles, and the absolute *gh*-expressing cell volume is significantly larger in adult females than juveniles or adult males. However, the relative volume of these populations remains stable. In contrast, *sl* mRNA levels are significantly higher in juveniles compared to adult males (*p* < 0.05), while the absolute cell volume tends to be larger (but not significantly) in adults than in juveniles. However, the relative volume of the *sl*-expressing cell population remains stable across sexes and stages.

Adult females have significantly higher mRNA levels of both *fshb* (*p* < 0.01) and *lhb* (*p* < 0.0001) compared to the other groups, which is consistent with the tendency of larger cell volume for both cell types. However, the relative volumes of these populations also remain stable. *tshba* mRNA levels are significantly higher in adult female (*p* < 0.0001), consistent with the significantly larger *tshba*-expressing cell absolute and relative volumes in the adult female pituitary. In contrast, *pomca* mRNA levels are significantly higher in adult male (*p* < 0.0001), in agreement with the increased absolute volume of the *pomca*-expressing cell population in adults compared to juveniles, which tends to be larger in adult males than in adult females. Furthermore, the relative volume of *pomca*-expressing cells is significantly larger in adult males than adult females.

Despite obvious differences in mRNA transcript levels and cell volume observed respectively with qPCR and FISH, we could not detect any differences in the proportions of each cell type between adult males and females by scRNA-seq.

### Cells Producing Multiple Hormones

**Figure 5:**
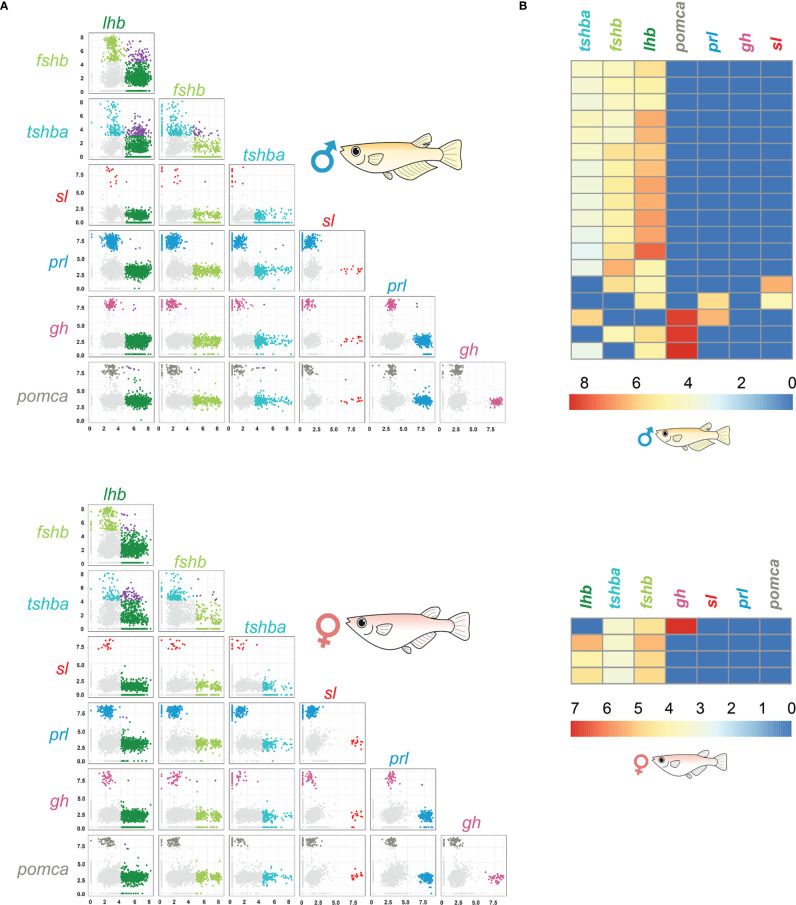
scRNA-seq data reveal the presence of bi-hormonal and multi-hormonal cells in the medaka pituitary. Pair wise plots of 2228 cells in female and 3245 cells in male pituitary **(A)**. Colored by filtered cells (light gray), lhb expressing cells (light green), fshb expressing cells (dark green), tshba expressing cells (cyan), sl expressing cells (red), prl expressing cells (blue), gh expressing cells (magenta), pomca expressing cells (dark grey) and cells expressing more than one endocrine gene (purple). Light grey cells represent the cells where gene expression for the investigated hormone is considered as part of the background. Axes are log normalized. Zoom in from the heatmap of seven hormone-encoding genes of the male and female pituitary shown in **Supplementary Figure 15**, displaying the cells expressing more than two hormone-encoding genes **(B)**. Each row represents one cell, and low expressions are shown in blue and high expressions are shown in red. For all hormone-encoding genes, expression levels below the threshold established in **Supplementary Figure 1** were replaced by zero and thus appear in blue.

**Figure 6:**
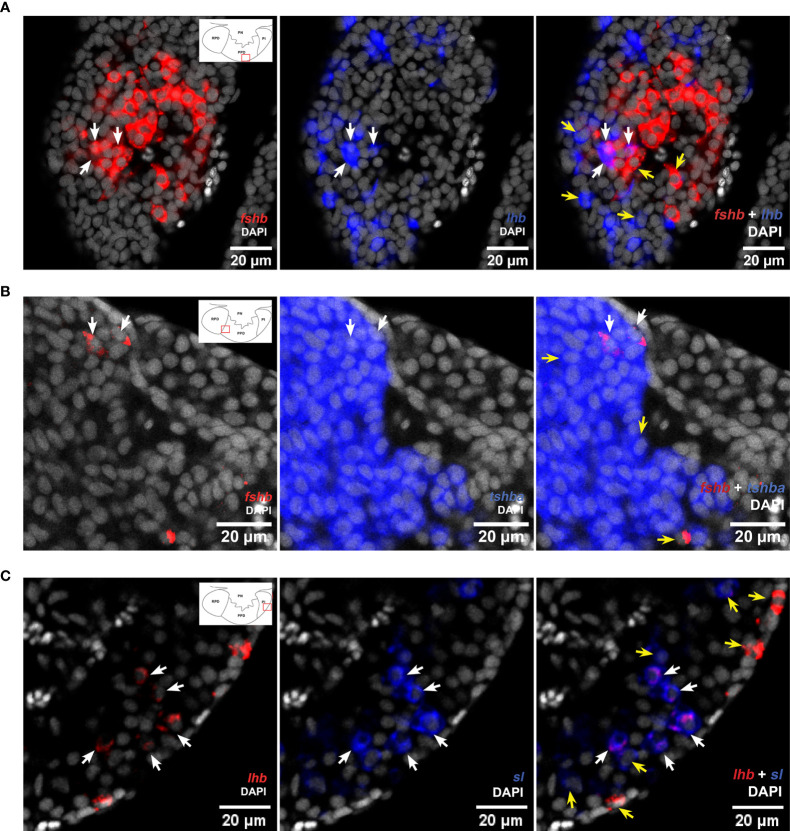
Multi-color FISH reveals some cells co-expressing more than one hormone-encoding genes in the medaka pituitary. Single confocal planes (pinhole aperture 1 Airy Unit (0.8 -1.1 µm section)) confirming colocalization of *lhb* and *fshb* **(A)**, and *fshb* and *tshb* **(B)** in both male and female, and *lhb* and *sl* in the adult male medaka pituitary **(C)**. White arrows show cells with co-expressed mRNAs, while yellow arrows show cells without (can be used as control of probe´s specificity). The location of the bi-hormonal cells is in the proximity of the red rectangles as illustrated in the schematic drawings of the pituitaries in left panels.

Using scRNA-seq, we identified 191 and 229 bi-hormonal cells in the adult female and male medaka pituitaries, respectively (**Figure 5A** and **Supplementary Table 1**). Both sexes show a number of cells co-expressing *lhb*- and *fshb*-, *lhb*- and *tshba*- and *fshb* and *tshba*. Meanwhile, some cells co-expressing *lhb* and *sl*, *fshb* and *sl*, *fshb* and *prl*, *fshb* and *pomca*, *tshba* and *prl*, *tshba* and *pomca*, *prl* and *gh*, and *prl* and *pomca* were unique to adult males, whereas cells co-expressing *fshb* and *gh* were only found in adult females. The existence of cells co-expressing *lhb*-*fshb* and *fshb*-*tshba* in both sexes and *lhb*-*sl* in adult male was confirmed using multi-color FISH (**Figure 6**). While the co-localization of *lhb*-*fshb* and *fshb*-*tshba* was observed in several individuals, the *lhb*-*sl* expressing cells were observed only in 1 of 13 adult male pituitaries analyzed. However, we could not detect colocalization between *tshba* and *lhb* by FISH.

We then investigated whether some of these bi-hormonal cells were expressing more than two hormone-encoding genes (**Figure 5B** and **Supplementary Figure 15**). We found a few cells co-expressing three hormone-encoding genes, although these are rare, representing only 0.15% and 0.47% of all pituitary cells in females and males, respectively. We did not detect any cell co-expressing more than three hormone-encoding genes. These multi-hormonal cells could not be detected using multi-color FISH.

## Discussion

### 3D Spatial Distribution of Endocrine Cell Populations and Blood Vessels

**Figure 7:**
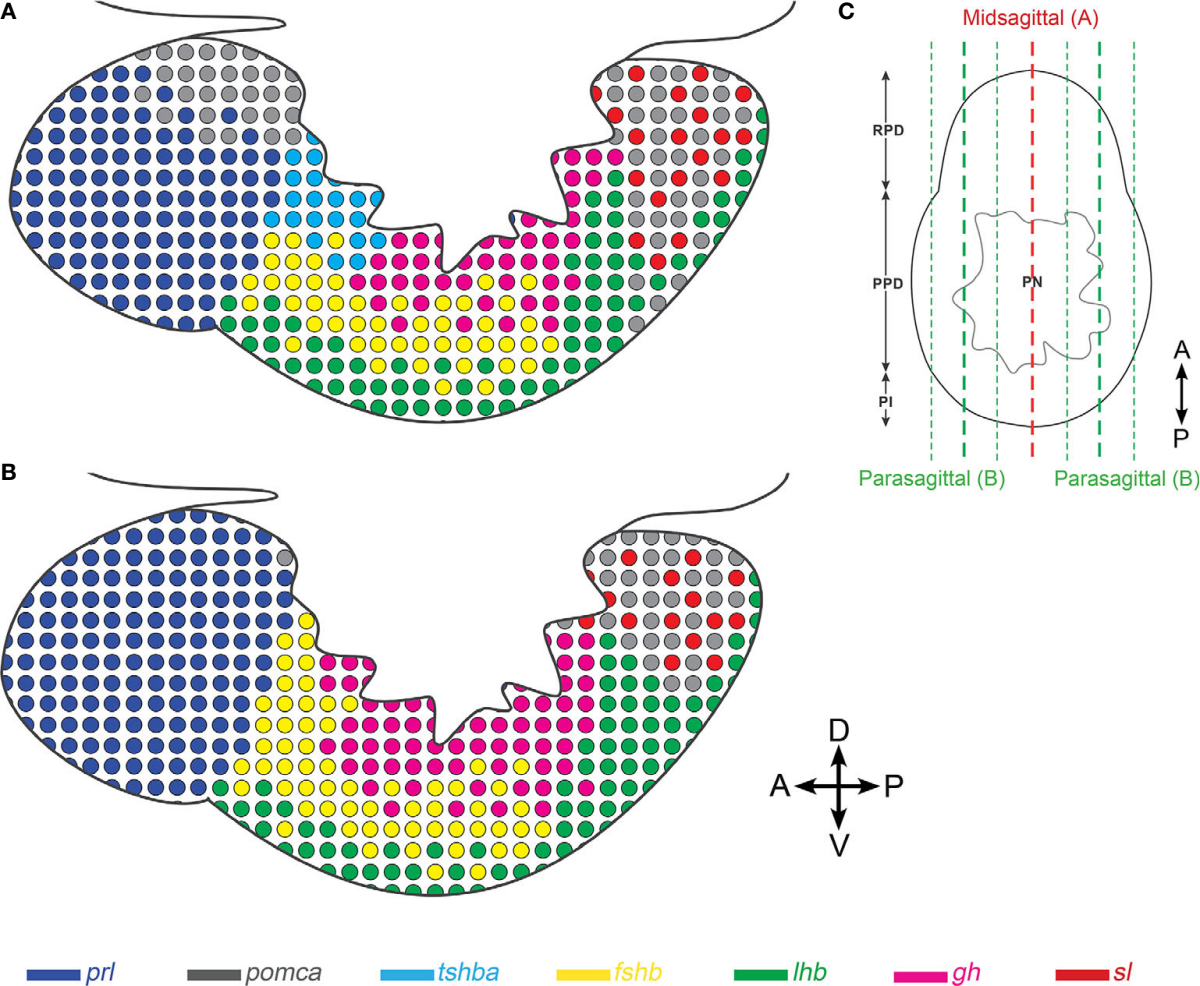
Schematic illustration showing differences in the distribution of endocrine cell populations between mid-sagittal and para-sagittal sections of the medaka pituitary. The schemas are drawn based on mid- **(A)** and para-sagittal **(B)** points of view. **(C)** Transverse view of medaka pituitary showing approximate location of mid-sagittal (red) and para-sagittal sections (green). Four direction arrows display the direction of the pituitary (A, anterior; P, posterior; D, dorsal; V, ventral).

We have recently used scRNA-seq to identify and characterize seven endocrine cell types in the teleost model organism medaka (30). Although a 3D atlas of the pituitary gland development has been previously described in zebrafish (42), the present atlas is the first 3D atlas of all pituitary endocrine cell populations in a teleost fish. It provides more precise and detailed information on the distribution and organization of the different cell types, and clearly demonstrates that endocrine cells are distributed differently in mid-sagittal versus para-sagittal sections (**Figure 7**).

As reported in coho (43) and chum salmon (44, 45), seabass (46), gilt-head seabream (47), common barbel (48) and striped bass (49), we observed some lactotropes in the ventro-peripheral area of the PPD. However, these cells were found only in some fish and not always at the same location, making them difficult to map. We found Lh gonadotropes in the PI, in addition to in the PPD, but only in adults, and we did not observe Lh cells in RPD as reported by some studies (15, 16, 50–52). The extra-PPD localization of Lh gonadotropes might be due to extension of the PPD into the PI (19) or to Lh cell migration to other zones during the ontogeny of the adenohypophysis (53). Meanwhile, several studies reported somatotropes in the RPD (49, 54) and PI (55, 56), and somatolactotropes and melanotropes in the teleost PPD (12, 15–17, 55). However, we did not observe this in medaka. The wide localization of Gh, Sl, and α-Msh cells in these immunohistochemical studies might also be explained by antigenic similarities as previously suggested (12, 15, 57).

The vasculature is ubiquitously spread throughout nearly the entire adenohypophysis in medaka, without obvious differences between sexes and stages. This agrees with previous studies in zebrafish (58, 59) showing a highly vascularized pituitary. Such complex vasculature is, of course, central to the endocrinological function of the pituitary, as it allows for the efficient transport of secreted hormones to peripheral organs. In addition, it may facilitate intra-pituitary signaling (26).

### Sexual Dimorphism of *tshba-, pomca-*, and *lhb*-Expressing Cell Populations

While studies using only para-sagittal pituitary sections were unable to resolve differences of endocrine cell population between sexes and stages, whole pituitary labeling methods allow us to detect such differences. For instance, we show for the first time that *tshba*-expressing cell volumes are the largest in adult females, and *pomca*-expressing cell volumes are the largest in adult males. Our qPCR data on *tshba* and *pomca* levels agree with a previous study in medaka (60) and further support the sexual dimorphism. In that study, androgens were shown to stimulate transcription of *tph1*, which encodes an enzyme required for serotonin synthesis, in *pomc*-expressing cells. The authors hypothesized that in males, higher androgen levels lead to higher serotonin levels, which repress the expression of some hormone-encoding genes including *tshb*. We also found significantly lower *tshba* levels in adult compared to juvenile males where the androgen levels are generally lower [for review, see (61, 62)], supporting the inhibitory role of androgens on *tshba* levels. It will be interesting to test this hypothesis in future research using orchidectomy which drastically reduce androgen levels (63). However, although higher *tshb* levels are also observed in female half-barred wrasse (64), and *tshb* levels are higher in juvenile than in adult male Atlantic salmon (65), zebrafish show no sexual dimorphism of *tshb* and *pomc* (66), suggesting species differences.

We also observed sexual dimorphism of *fshb* and *lhb* mRNA levels in adults, in agreement with a previous medaka study (60). Although *lhb*-expressing cell volume also shows sexual dimorphism, we do not observe this for *fshb*-expressing cells. This suggests a difference in gonadotrope cell activity, as supported by the absence of significant differences in Fsh cell numbers in a previous study (8). In contrast, we observed an increase of *lhb* mRNA levels from juvenile to adult stages, which might be due to increased cell numbers as previously reported (41). Surprisingly, we did not observe a significant difference in *fshb* mRNA levels between juveniles and adults, despite a previous report of an increase in Fsh cell numbers during sexual maturation (8). While neither sexual dimorphism nor stage differences were observed for *prl* or *gh* levels, we found a stage effect on the absolute volume of *prl*-expressing cells, and sexual dimorphism of the absolute volume of *gh*-expressing cells in adults. The latter finding is consistent with a previous study that showed sexual dimorphism of *gh* levels in adult medaka (60), while previous studies in blue gourami (67) and gilthead seabream (68) contrast with our findings on *prl* levels, suggesting species differences. Meanwhile, although *sl*-expressing cell volumes tend to be larger in adults than juveniles, we found the opposite for the mRNA levels, with higher levels in juveniles than in adults, suggesting higher cell activity in juveniles. Indeed, somatolactin has been associated with sexual maturation in some teleosts, such as coho salmon (69), Nile tilapia (70) and flathead grey mullet (71). The upregulation of *sl* levels in teleosts is thought to be related to gonadal growth, as it is highly expressed at the onset of gonadal growth and lowly expressed post-ovulation (72, 73). This implies that in the current study, the adult fish used may have been in a post-ovulation phase while the juveniles may have been initiating gonadal development.

### scRNA-seq and Multi-Color FISH Reveal the Presence of Multi-Hormonal Cells in the Adult Medaka Pituitary

The presence of cells expressing more than one hormone in the anterior pituitary has been shown in many studies, both in teleosts and in mammals [for review see (27, 74–76)]. Using scRNA-seq technology, multi-hormonal cells have been described in the mouse pituitary (77), but never before in a teleost. While scRNA-seq has previously been used to analyze the zebrafish pituitary, the existence of multi-hormonal cells was not investigated (78). Using similar approaches, we previously could not identify any clusters of multi-hormonal cells in the medaka pituitary (30). Thus, in the present study, we more deeply analyzed our scRNA-seq data and found a relatively small number of bi-hormonal and very few multi-hormonal cells. Such low numbers of multi-hormonal cells could explain the lack of detection of cell clusters in the previous medaka study (30).

In the current study, we show the presence of gonadotrope cells co-expressing *lhb* and *fshb* which has previously been reported in medaka (8) and in other teleost species (7, 9, 10). We also found cells co-expressing *fshb-tshba* and *sl*-*lhb*. Although previous immunohistochemtry studies on the pituitary of several teleost species showed cross-reaction between Tsh and Lh/Fsh (47, 57) and between Lh and Sl antibodies (15, 57, 79, 80), we show discrete labeling of *tshba*, *fshb*, *lhb*, and *sl* cells in the current study confirming the specificity of the probes. Therefore, the observation of colocalization of *fshb-tshba* and *sl*-*lhb* supports that these bi-hormonal cells exist in the medaka pituitary. Meanwhile, several studies have shown co-staining between Prl, Gh and Sl (12, 19, 21, 44, 55). However, our analysis of scRNA-seq data revealed only a few cells co-expressing *prl* and *gh*. While antigenic similarities could explain co-staining between these cell types in previous studies (12, 15, 57), their low occurrence in the medaka pituitary might prevent their identification with FISH. We also show that very few cells in the adult medaka pituitary express more than two hormone-encoding genes. The current study is the first to show the presence of such multi-hormonal cells in the teleost pituitary. However, it differs noticeably from mammals, where multi-hormonal cells are numerous and thus form a cluster in the scRNA-seq data (77), which is not the case in medaka (30). Although we could not confirm their existence using FISH, most likely because of their low incidence, the evidence of their existence using scRNA-seq raises questions about their origin and roles in the teleost pituitary.

In mammals, the presence of multi-hormonal cells has been described and associated with pituitary plasticity where the cell number is changing to fulfill physiological demands [for review, see (75)]. Hypothetical origins of these cells have been previously discussed (27, 74, 75, 81–85). They may originate from differentiating progenitor cells where a non-fully differentiated transient state could allow the expression of several hormone-encoding genes. This hypothesis is supported in mammals by the identification of a cluster of multi-hormonal cells expressing PROP1, a progenitor cell marker (77). However, there is no such evidence yet found in teleosts, as we could not find such a cluster in the medaka pituitary in the previous (30) and current studies. Indeed, we found only very few cells expressing more than two hormone-encoding genes, with a maximum of three hormone-encoding genes expressed. A previous study in medaka also demonstrated that gonadotropes do not appear as bi-hormonal cells but as either Lh or Fsh during early development (8), suggesting that progenitor cells might not be multi-hormonal in the teleost pituitary. In addition, multi-hormonal cells may also appear during trans-differentiation (when one cell changes phenotype). Indeed, a study in medaka showed that *fshb*-expressing cells could start to express *lhb in vitro*, becoming at least temporarily bi-hormonal (8). Interestingly, most of the multi-hormonal cells in our scRNA-seq data express *lhb*/*fshb*, *lhb*/*tshba* or *fshb*/*tshba*. In mammals, differentiation of progenitor cells to both gonadotropes and thyrotropes requires the transcription factor *Gata2* (86, 87), but co-expression with *Sf1* leads to gonadotropes whereas co-expression with *Pit1* leads to thyrotropes (88–91). In contrast, relatively little is known regarding pituitary cell lineage in teleosts. Nevertheless, it is plausible that trans-differentiation occurs between the gonadotrope and thyrotrope cell types in teleosts, as previously described in mammals (92).

Within the *pomca*-expressing cell population, we observed Acth staining alone at the border of the RPD/PPD and staining of both Acth and α-Msh in the PI. Co-staining between Acth and α-Msh is not uncommon as Acth-immunoreactive cells have been found in RPD and PI areas in other teleost species (7, 17, 19–21, 46, 47, 93). However, it must be noted that the target antigen of anti-Acth used in this study and most previous studies contains the target antigen for anti-α-Msh (https://www.uniprot.org/uniprot/P01189#PRO_0000024970). This might explain why we found both Acth and α-Msh cells in the PI. A previous study pre-incubating anti-Acth with α-Msh antigen demonstrated that Acth cells are localized in the RPD while α-Msh cells are found in the PI (20). This might also be the case in medaka.

Finally, the 3D atlas platform that is provided online will help research community to take a close look on the spatial distribution of endocrine cells and the vascularization of blood vessels in the pituitary.

## Data Availability Statement

The datasets presented in this study can be found in online repositories. The single-cell transcriptomics data can be accessed through the project accession number GSE162787 at the NCBI Gene Expression Omnibus (GEO: https://ncbi.nlm.nih.gov/geo/). The imaging data can be found at the NMBU open research data (https://doi.org/10.18710/NOGJQ2).

## Ethics Statement

The animal study was reviewed and approved by Norwegian University of Life Sciences.

## Author Contributions

RF and F-AW conceptualized and planned the work. RF, MP, JB, and F-AW obtained funding. NR did all cloning. MR and RF performed the experiments and acquired the imaging data. MR and GC processed the imaging data and developed the online 3D model, supervised by MP and JB. CH and KS analyzed the single cell transcriptome data. MR, CH, and RF wrote the paper with the inputs from all authors. All authors contributed to the article and approved the submitted version.

## Funding

This study was funded by the Norwegian University of Life Sciences (to RF) and the Norwegian Research Council grants No. 251307, 255601, and 248828 (to F-AW). The tools development received support from the European Union’s Horizon 2020 Framework Programme for Research and Innovation under the Specific Grant Agreement No. 945539 (Human Brain Project SGA3) (to JB) and the Research Council of Norway under Grant Agreement No. 269774 (INCF Norwegian Node) (to JB).

## Conflict of Interest

The authors declare that the research was conducted in the absence of any commercial or financial relationships that could be construed as a potential conflict of interest.

## Publisher’s Note

All claims expressed in this article are solely those of the authors and do not necessarily represent those of their affiliated organizations, or those of the publisher, the editors and the reviewers. Any product that may be evaluated in this article, or claim that may be made by its manufacturer, is not guaranteed or endorsed by the publisher.

## Supplementary Material

Supplementary Figure 1 Density plots of seven hormone-encoding genes in the pituitary of adult male **(A)** and female **(B)** medaka. Red line represents a cut-off to differentiate between the cells with high gene expression (considered as endocrine cells) and low gene expression (considered as background and non-endocrine cells). X-axis represents the expression level on a logarithmic scale.

Supplementary Figure 2–8 Orthogonal views of each endocrine cell types in juvenile and adult male and female medaka. (2, *prl*; 3, *pomca*; 4, *tshba*; 5, *fshb*; 6, *lhb*; 7, *gh*; 8, *sl*). The pictures were captured from different perspectives: transverse (i), frontal (ii) and para-sagittal (iii). Up down arrow symbol shows the direction of the pituitary (A, anterior; P, posterior; D, dorsal; V: ventral).

Supplementary Figure 9–13 Transverse views of multi-color FISH of endocrine cells in juvenile and adult male and female medaka. (9, *prl*-*tshba*-*pomca*; 10, *sl*-*lhb*-*pomca*; 11, *fshb*-*tshba*; 12, *fshb*-*lhb*; 13, *fshb*-*gh*) (A, anterior; P, posterior; D, dorsal; V, ventral).

Supplementary Figure 14 The combination of FISH for *pomca* and IF for Acth or α-Msh allows the distinction of two clear *pomca*-expressing cell populations in the juvenile medaka pituitary. The distinction of Acth (green) and α-Msh (blue) producing cells from *pomca*-labelled cells (red) in the pituitary from juvenile male and female medaka. Dashed lines represent the pituitaries shown in the right panel. Four direction arrows display the direction of the pituitary (A, anterior; P, posterior; D, dorsal; V, ventral).

Supplementary Figure 15 Heatmap of seven hormone-encoding genes from the 229 and 191 bi-hormonal cells found in the adult male and female medaka pituitaries, respectively. Each row represents one cell. Low expressions are shown in blue and high expressions are shown in red. All expression levels below the threshold established in **Supplementary Figure 1** were replaced by zero values.

Supplementary Table 1 Percentage of cells expressing two hormone-encoding genes in male and female pituitary scRNA-seq data. For instance, when looking at *lhb*- and *fshb*-expressing cells in males, we can observe that 72.6% of the cells express *lhb* but not *fshb*, 23.4% express *fshb* but not *lhb* while 4% express both *fshb* and *lhb*. The bi-hormonal *lhb/fshb* cells represent 1.79% of all pituitary cells. Empty spaces mean that no bi-hormonal cells were found.
