# Supplementary material for: 3D Atlas of the Pituitary Gland of the Model Fish Medaka (Oryzias latipes)
Source: Front Endocrinol (Lausanne). 2021 Aug 23;12:719843. doi: 10.3389/fendo.2021.719843 (PMC8419251; doi:10.3389/fendo.2021.719843)
Supplement: Supplementary Figure 1 — Density plots of seven hormone-encoding genes in the pituitary of adult male (A) and female (B) medaka. Red line represents a cut-off to differentiate between the cells with high gene expression (considered as endocrine cells) and low gene expression (considered as background and non-endocrine cells). X-axis represents the expression level on a logarithmic scale. [file DataSheet_1.pdf]

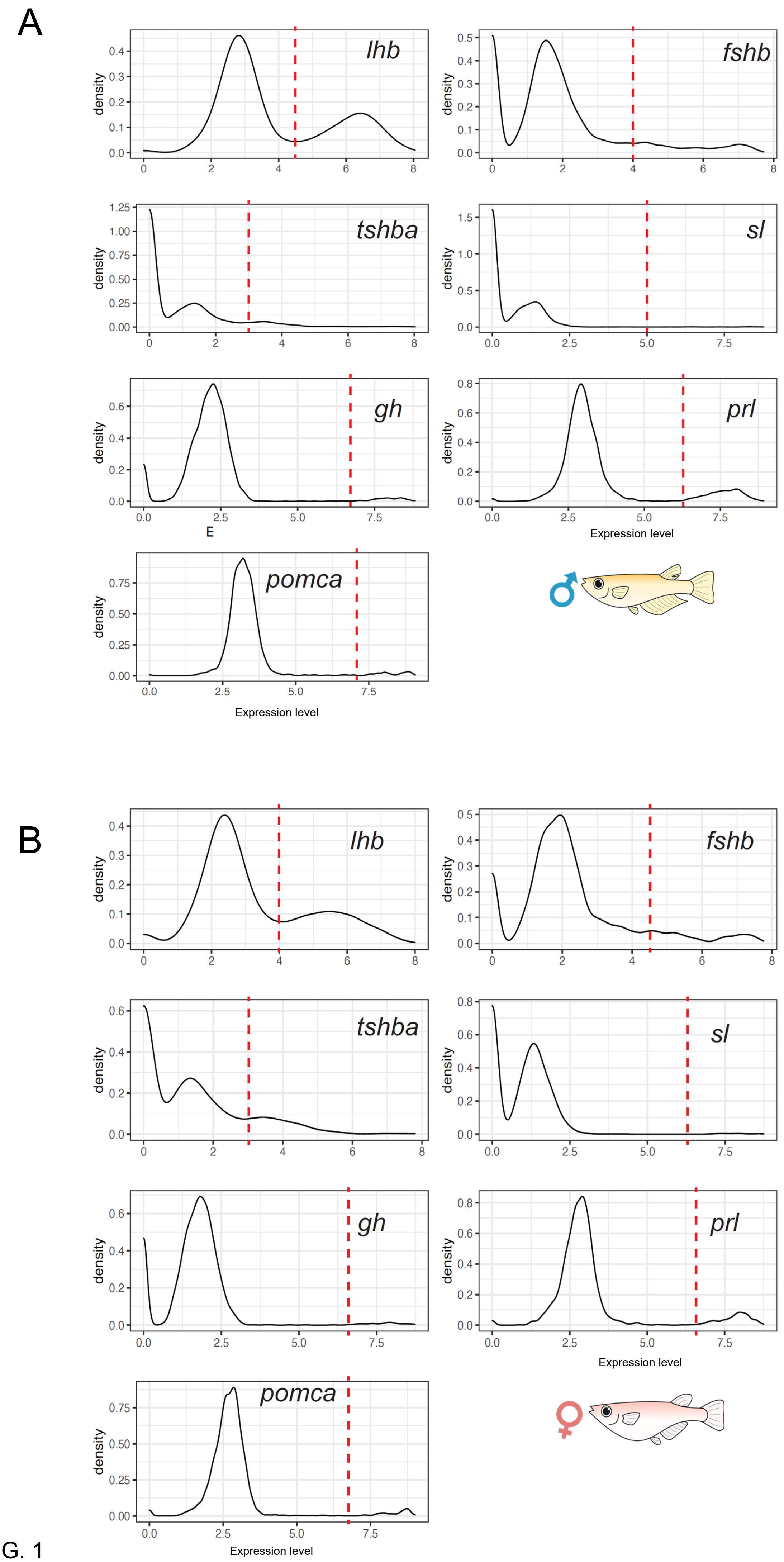
A
lhb
fshb
tshba
sl
gh
prl
pomca
density
E
Expression level
♂
B
lhb
fshb
tshba
sl
gh
prl
pomca
density
Expression level
♀

SUPP. FIG. 1

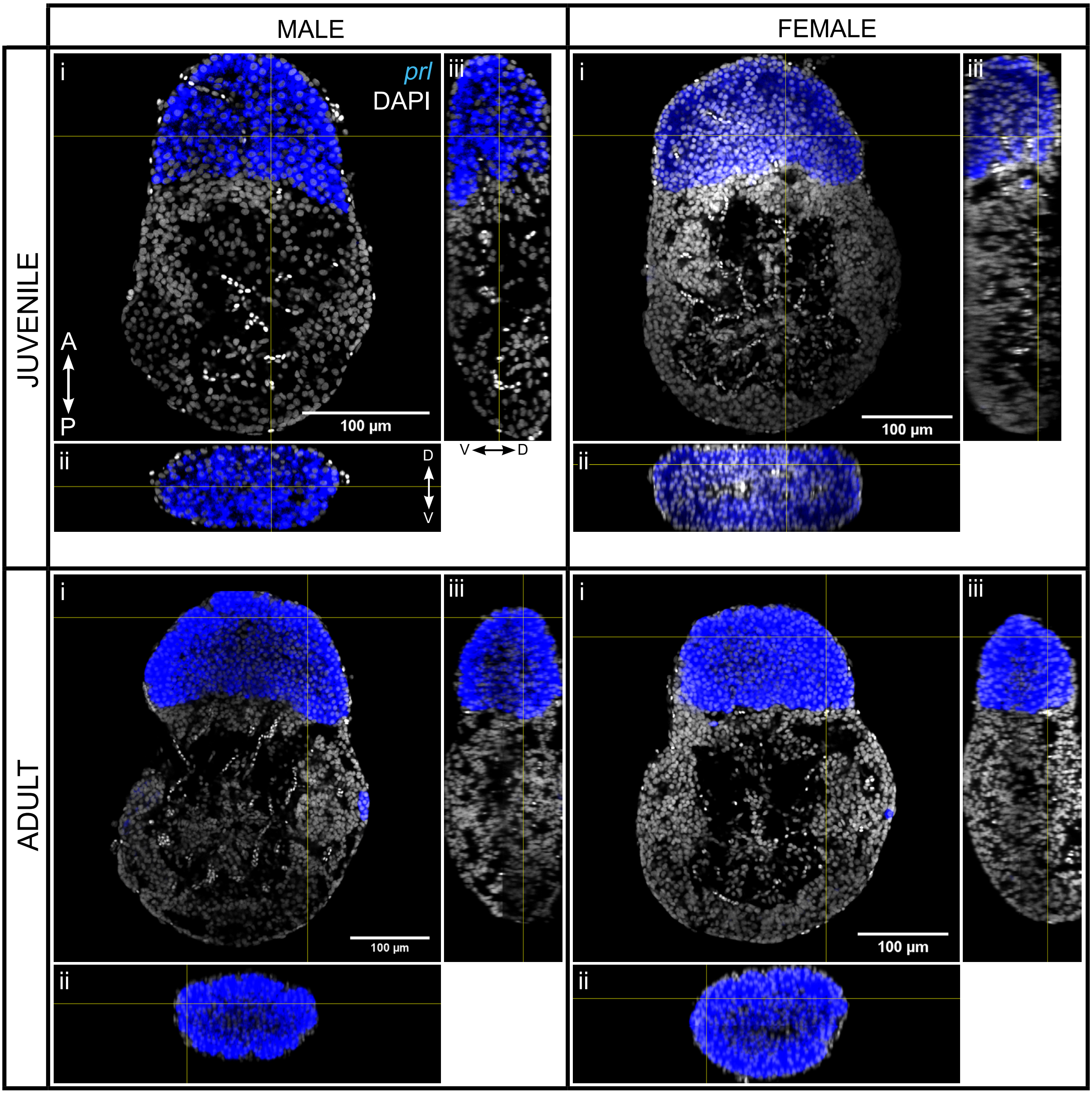

SUPP. FIG. 2

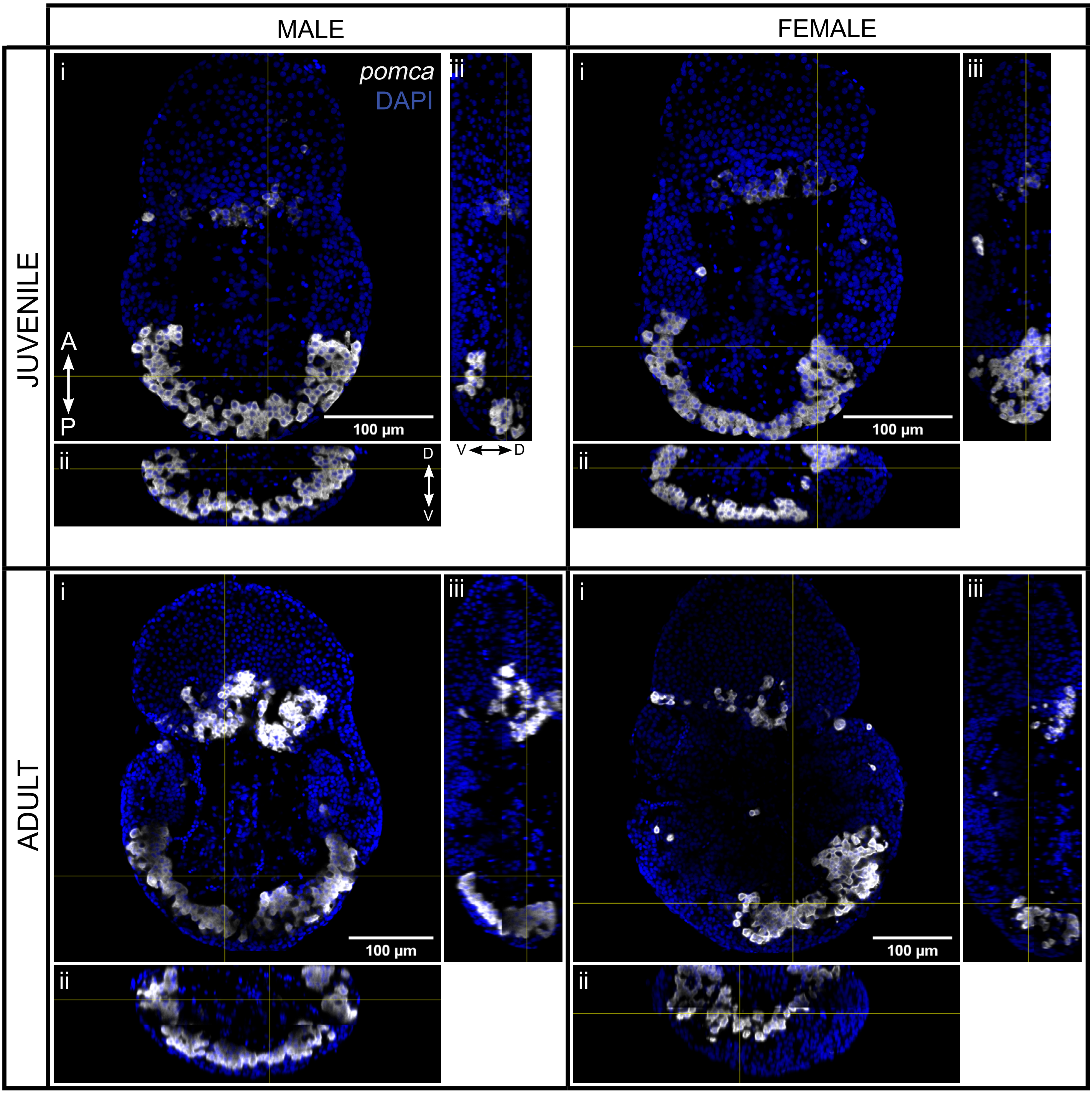

SUPP. FIG. 3

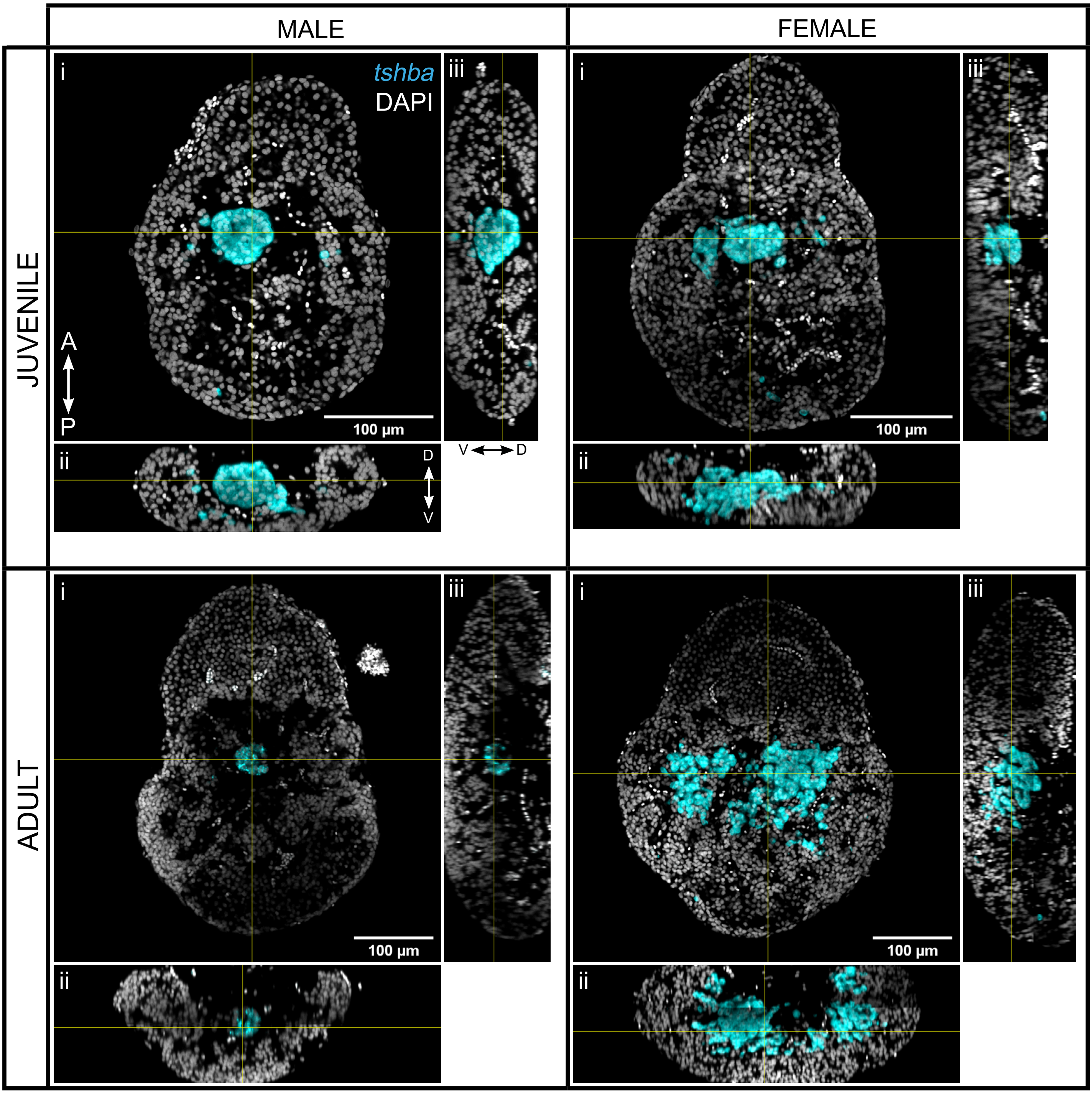

SUPP. FIG. 4

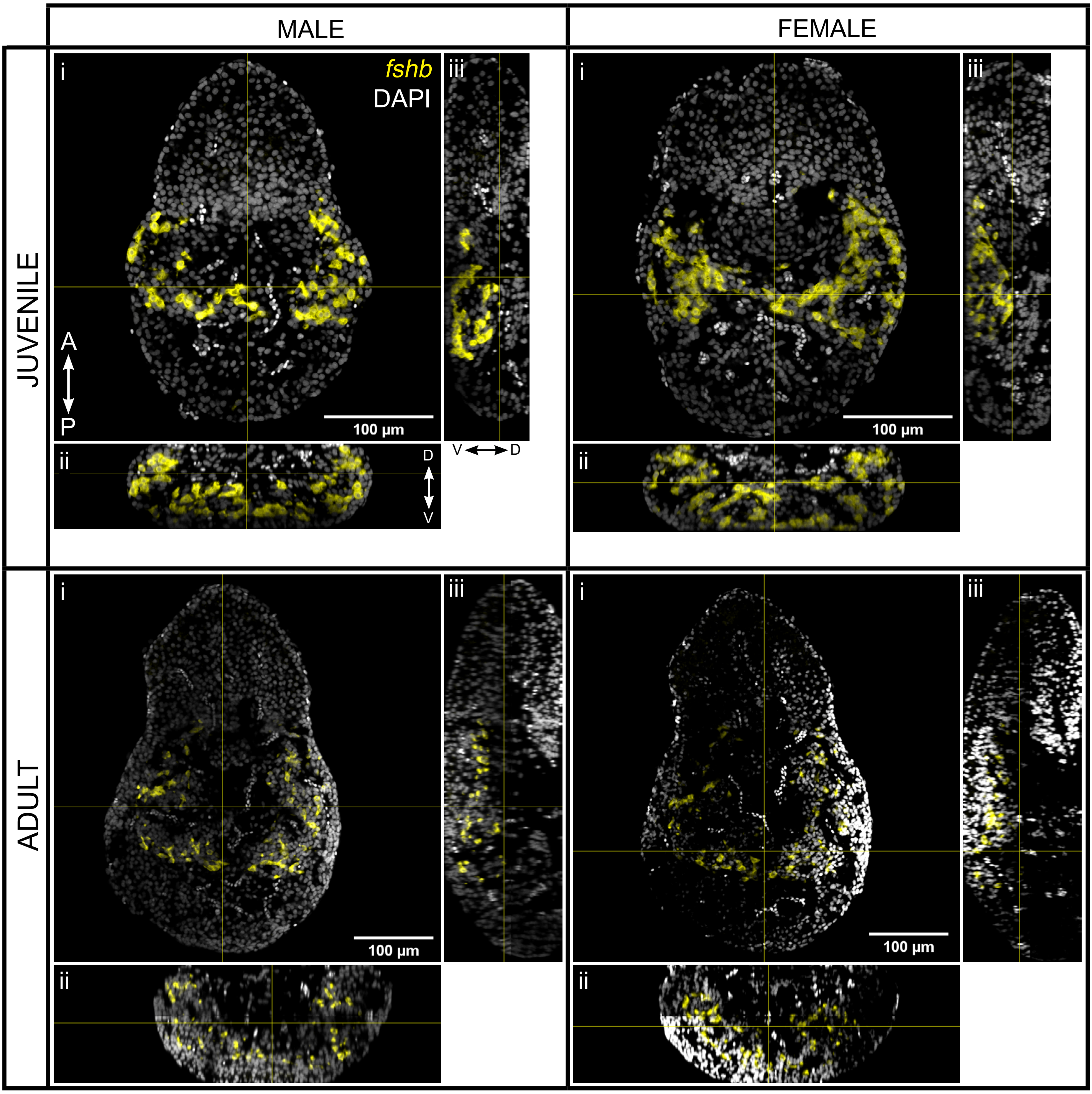
MALE
FEMALE
JUVENILE
ADULT
i
ii
iii
fshb
DAPI
A
P
D
V
V
D
100 µm
100 µm
100 µm
100 µm

SUPP. FIG. 5

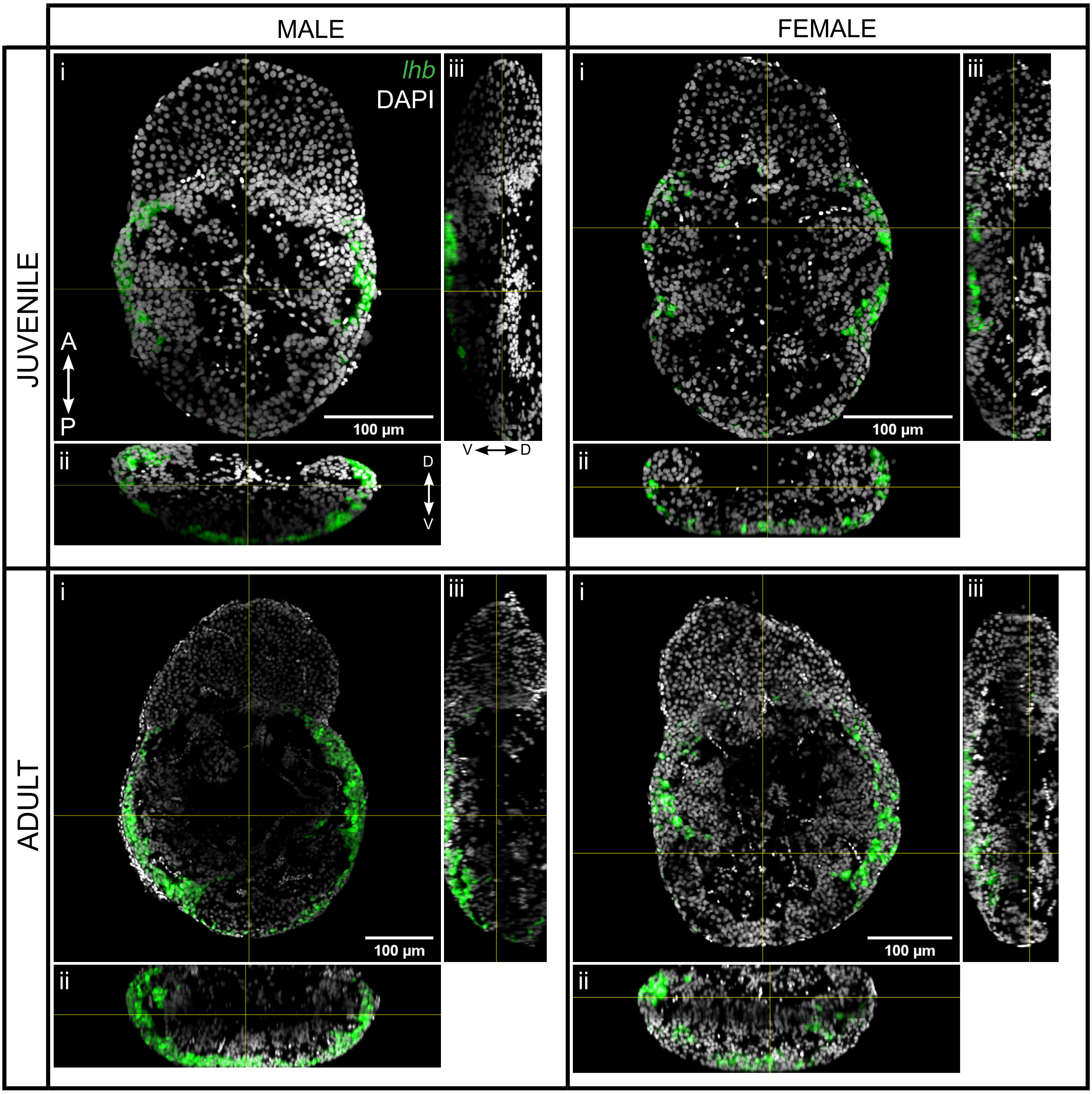

SUPP. FIG. 6

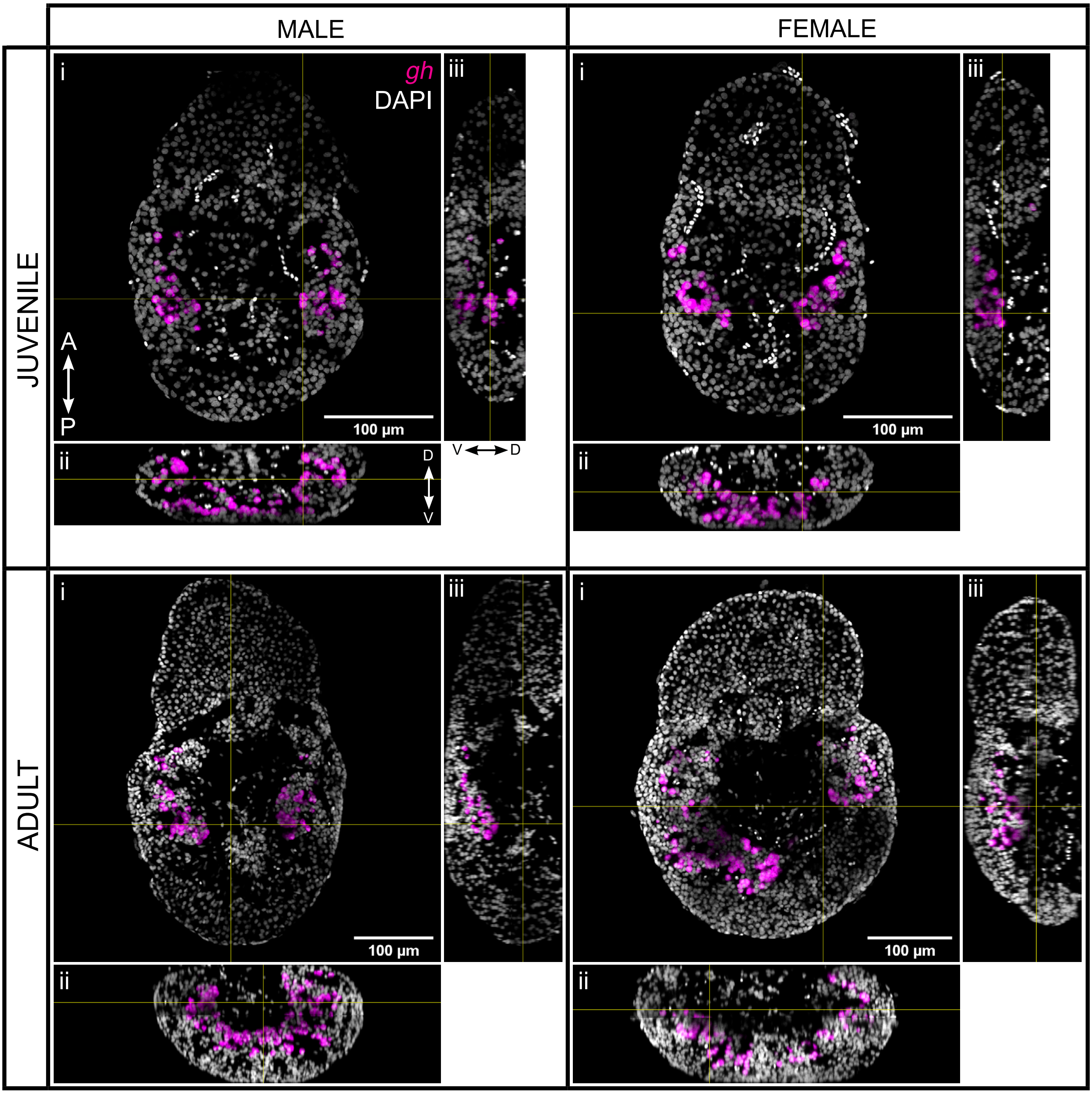

SUPP. FIG. 7

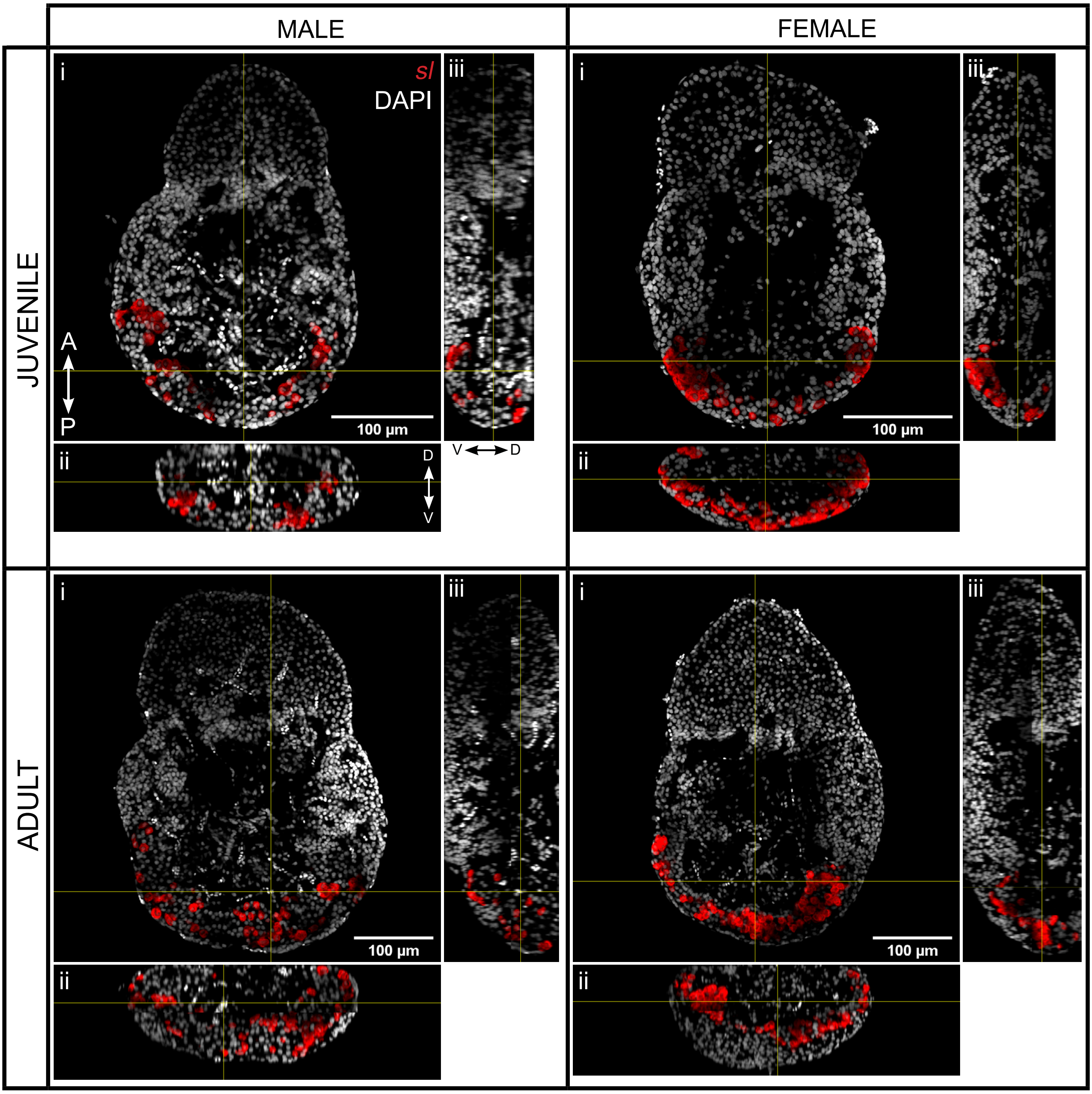

SUPP. FIG. 8

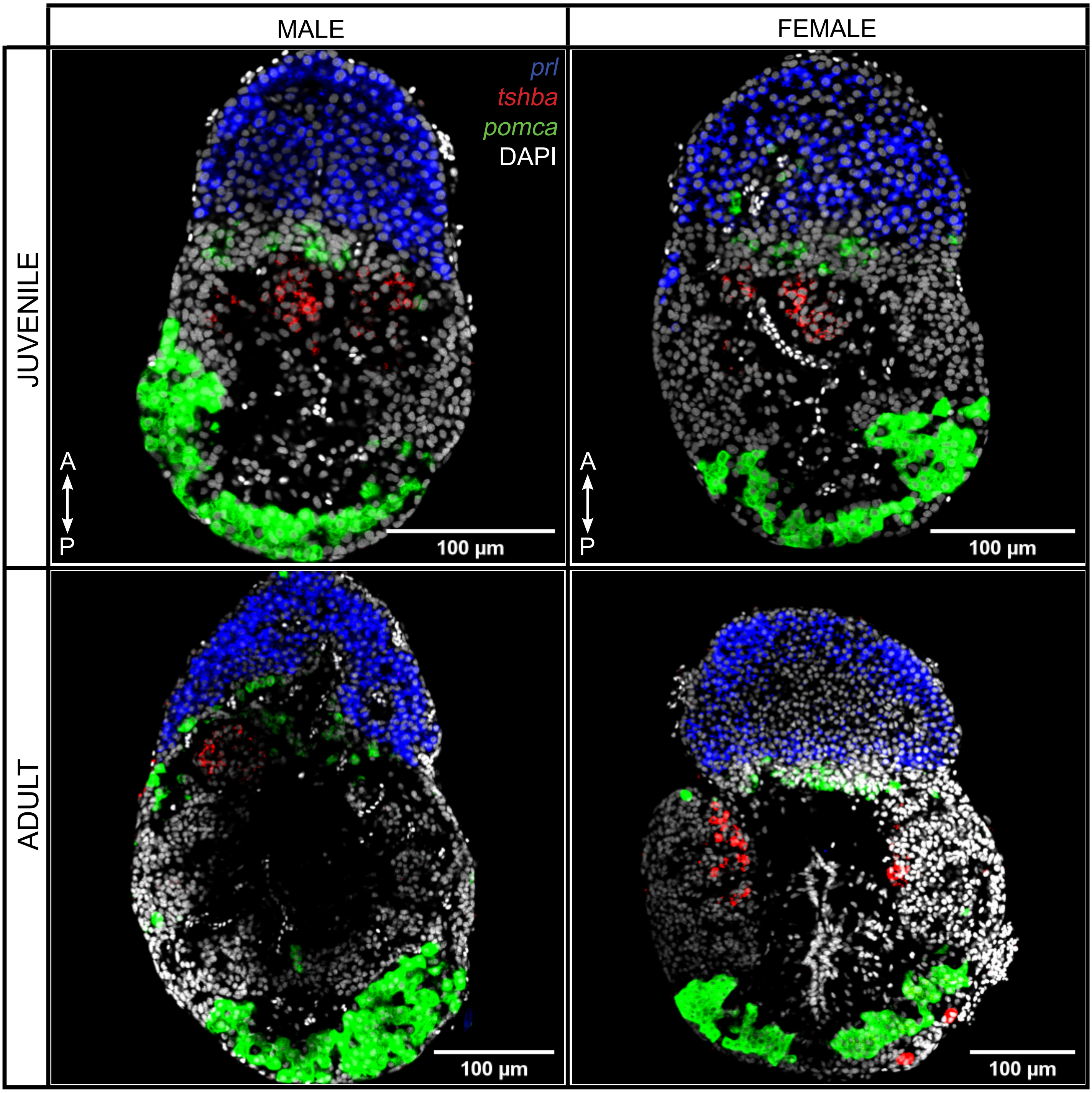
MALE
FEMALE
JUVENILE
ADULT
prl
tshba
pomca
DAPI
A
P
100 µm
A
P
100 µm
100 µm
100 µm

SUPP. FIG. 9

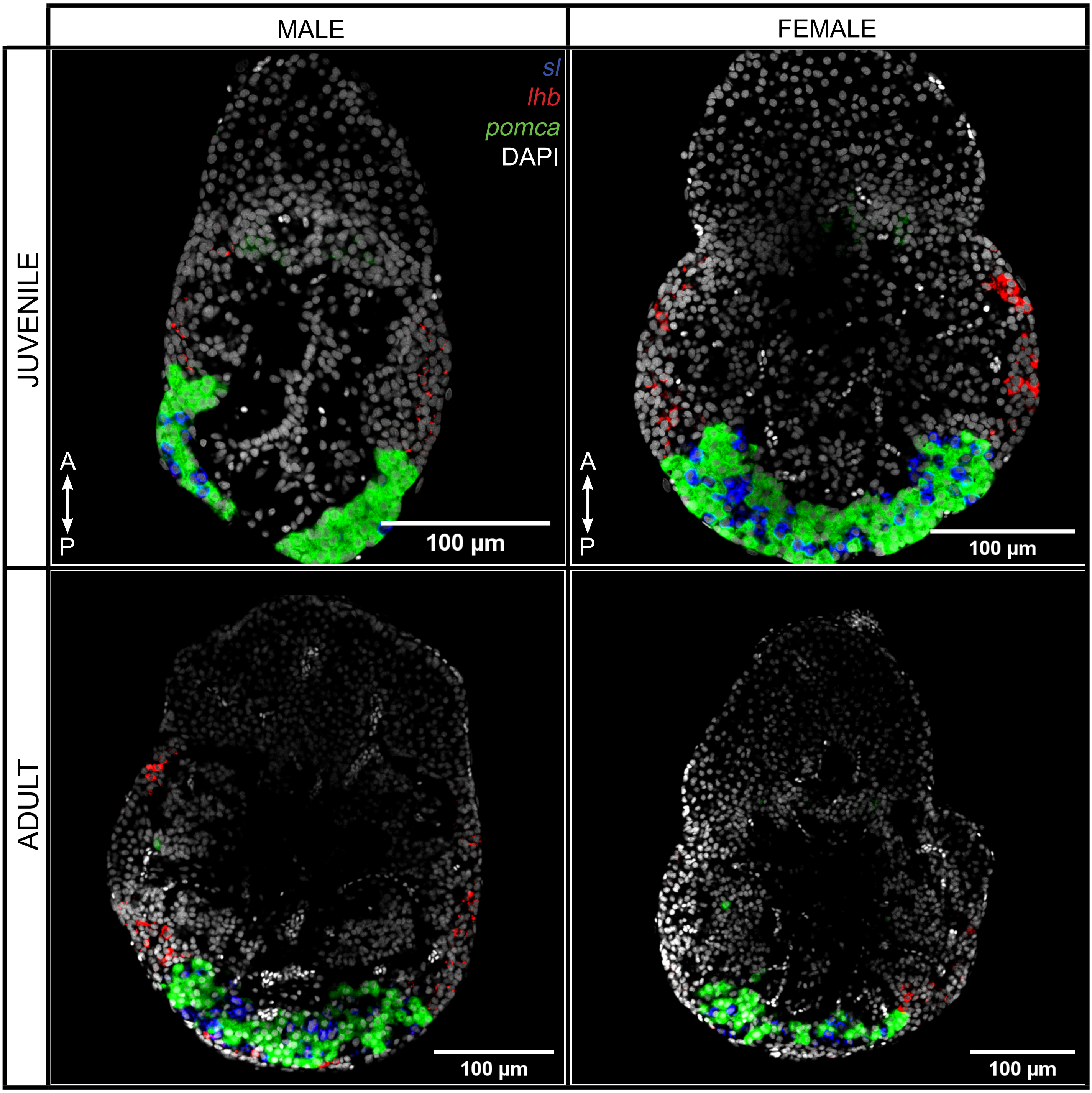
MALE
FEMALE
JUVENILE
ADULT
sl
lhb
pomca
DAPI
A
P
A
P
100 µm
100 µm
100 µm
100 µm

SUPP. FIG. 10

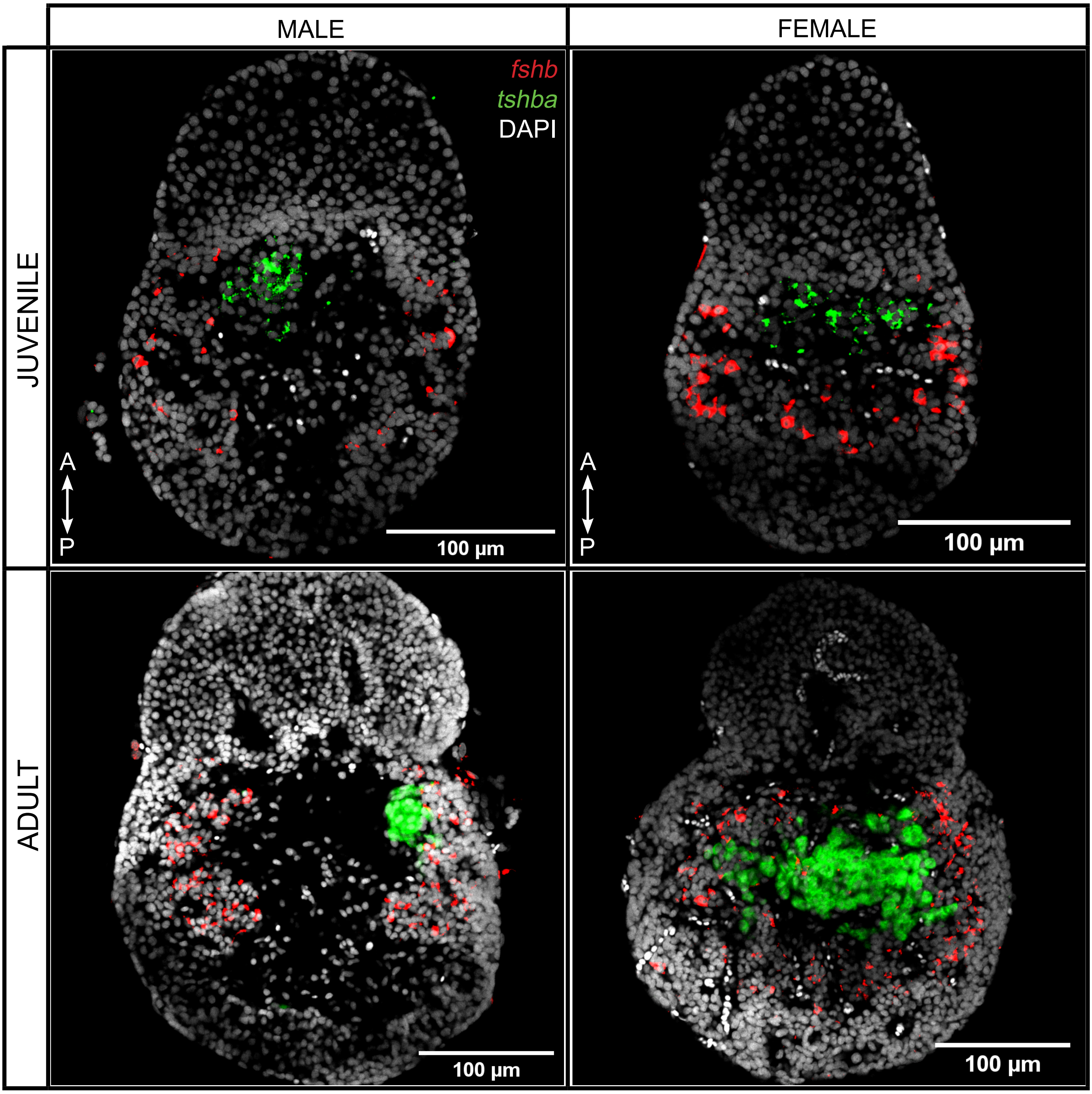
MALE
FEMALE
JUVENILE
ADULT
fshb
tshba
DAPI
A
P
100 µm
A
P
100 µm
100 µm
100 µm

SUPP. FIG. 11

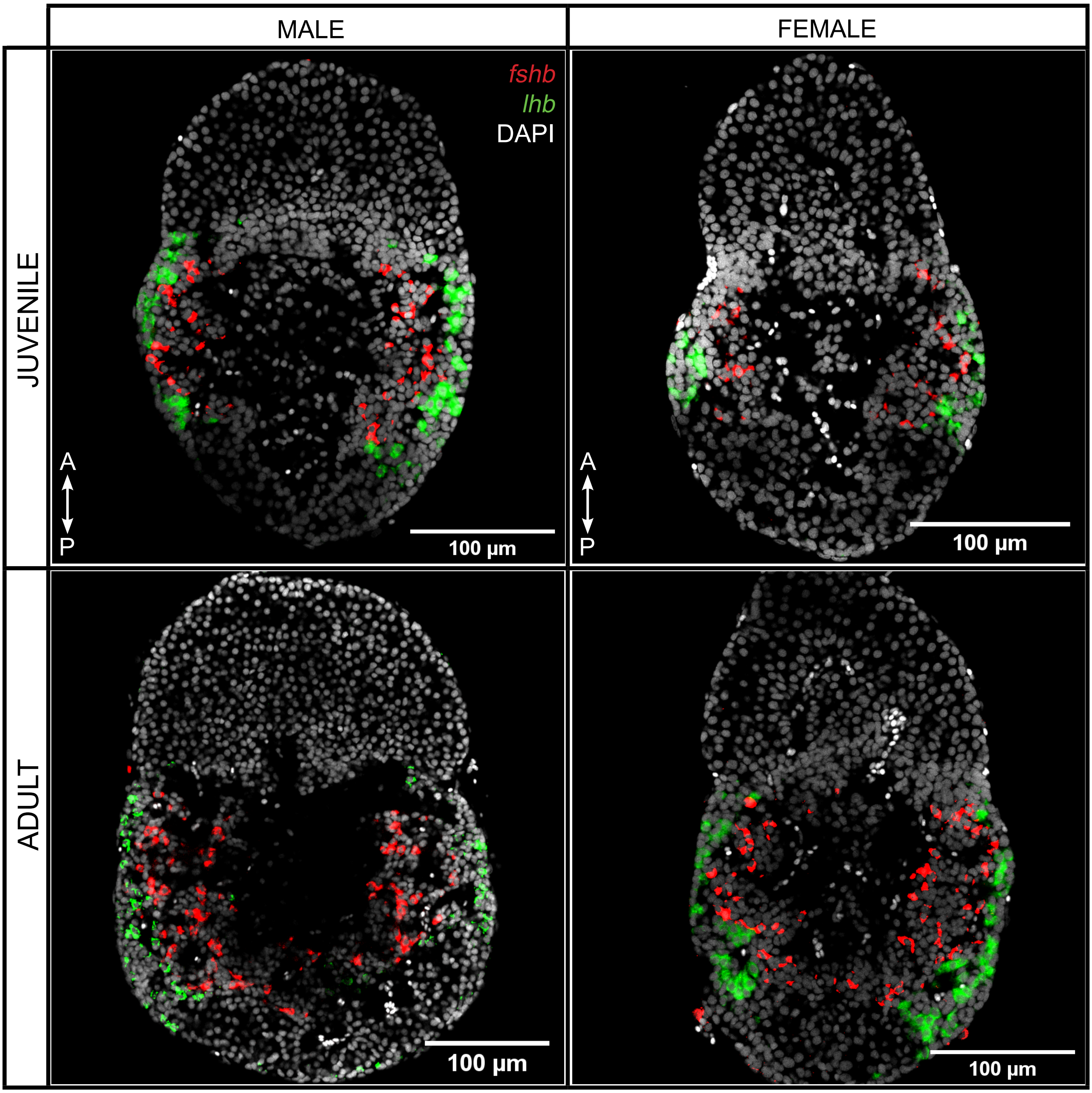
MALE
FEMALE
JUVENILE
ADULT
fshb
lhb
DAPI
A
P
A
P
100 µm
100 µm
100 µm
100 µm

SUPP. FIG. 12

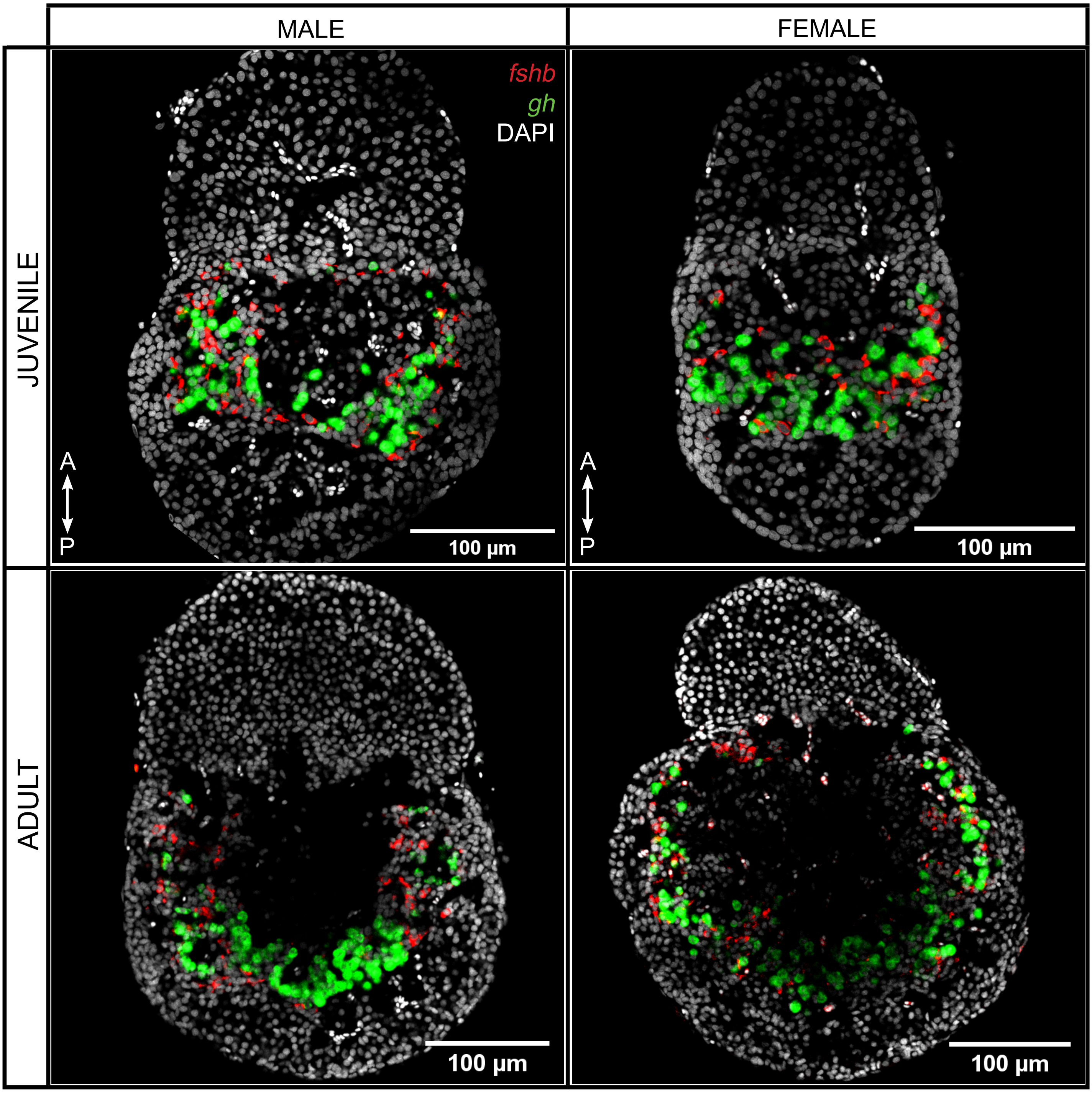
MALE
FEMALE
JUVENILE
ADULT
fshb
gh
DAPI
A
P
100 µm
A
P
100 µm
100 µm
100 µm

SUPP. FIG. 13

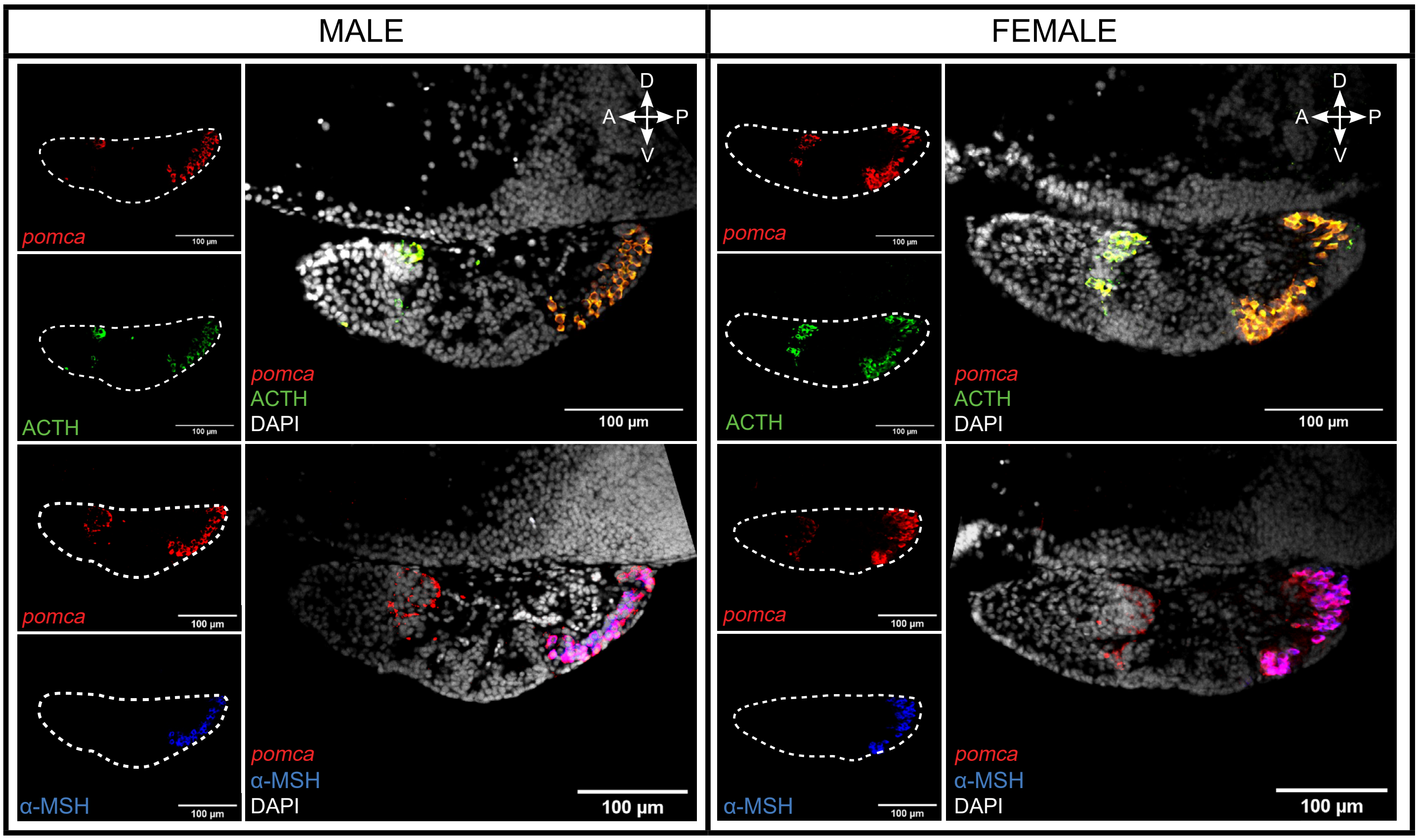

SUPP. FIG. 14

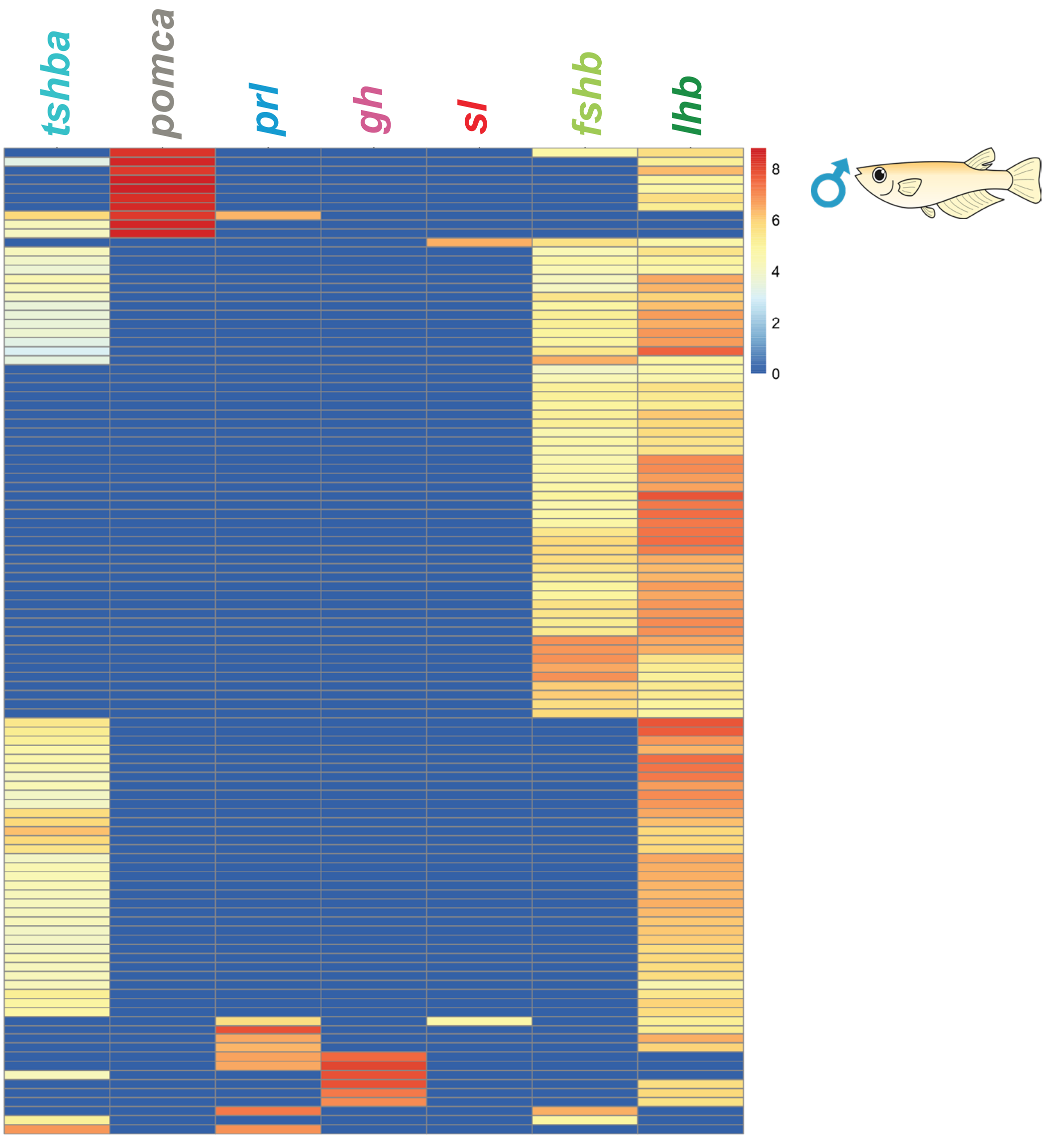

tshba
pomca
prl
gh
sl
fshb
lhb
8
6
4
2
0

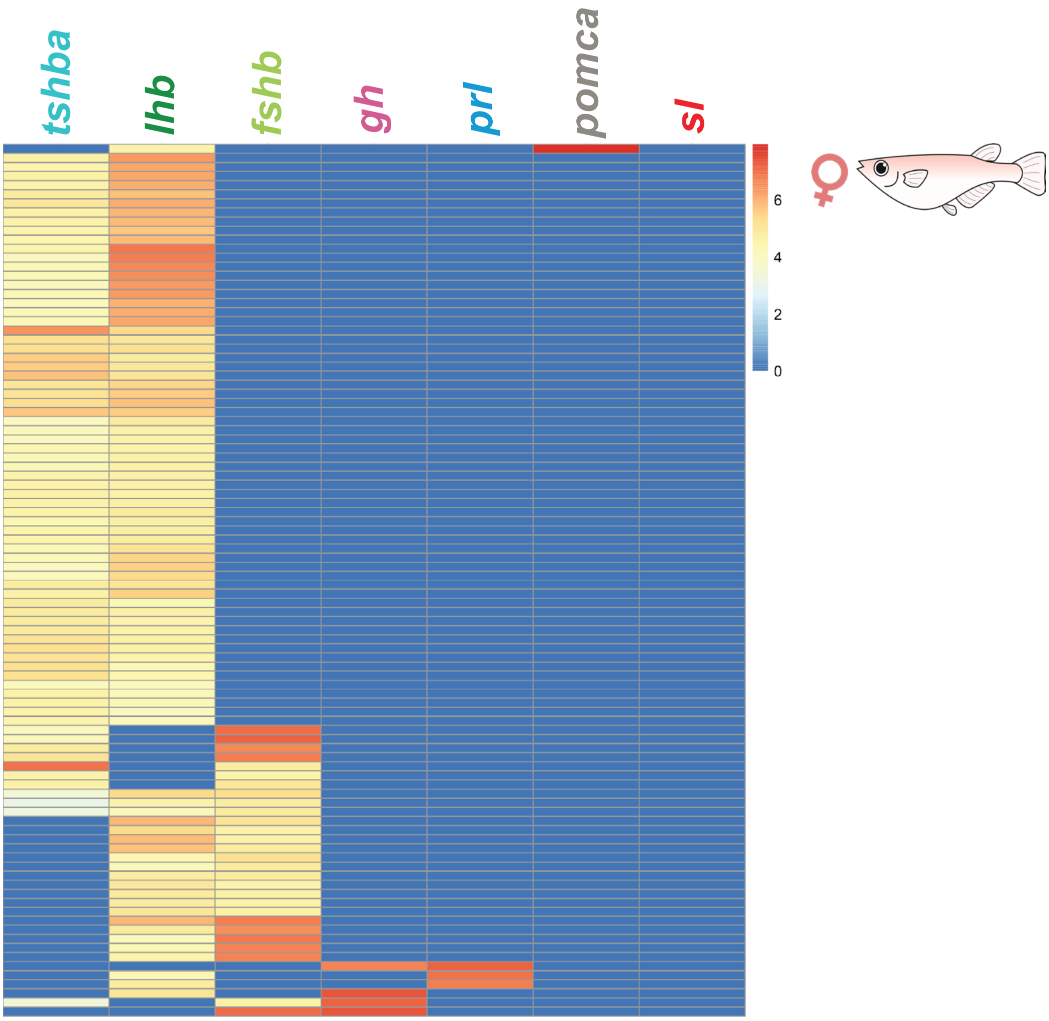

tshba
lhb
fshb
gh
prl
pomca
sl
6
4
2
0

SUPP. FIG. 15
