# Supplementary material for: 3D Atlas of the Pituitary Gland of the Model Fish Medaka (Oryzias latipes)
Source: Front Endocrinol (Lausanne). 2021 Aug 23;12:719843. doi: 10.3389/fendo.2021.719843 (PMC8419251; doi:10.3389/fendo.2021.719843)
Supplement: Supplementary Table 1 — Percentage of cells expressing two hormone-encoding genes in male and female pituitary scRNA-seq data. For instance, when looking at lhb- and fshb-expressing cells in males, we can observe that 72.6% of the cells express lhb but not fshb, 23.4% express fshb but not lhb while 4% express both fshb and lhb. The bi-hormonal lhb/fshb cells represent 1.79% of all pituitary cells. Empty spaces mean that no bi-hormonal cells were found. [file DataSheet_2.pdf]

Gene investigated		Male			Female		
(1)	(2)	(1)	(2)	Bi-hormonal	(1)	(2)	Bi-hormonal
lhb	*fshb*	72.6	23.4	4.0 (1.79)	74.0	23.9	2.1 (0.86)
lhb	*tshba*	88.2	8.5	3.3 (1.20)	75.9	16.7	7.4 (2.84)
lhb	*sl*	98.6	1.3	0.1 (0.03)			
lhb	*prl*	72.0	27.0	1.0 (0.49)	77.5	22.3	0.2 (0.09)
lhb	*gh*	91.8	7.8	0.4 (0.12)	93.8	6.0	0.2 (0.05)
lhb	*pomca*	90.7	8.4	0.9 (0.34)	89.6	10.3	0.1 (0.05)
fshb	*tshba*	76.6	23.0	0.4 (0.06)	58.3	39.8	1.9 (0.32)
fshb	*sl*	95.8	3.9	0.3 (0.03)			
fshb	*prl*	46.1	53.6	0.3 (0.06)			
fshb	*gh*				82.7	16.5	0.8 (0.09)
fshb	*pomc*	77.5	22.0	0.5 (0.06)			
tshba	*sl*						
tshba	*prl*	20.4	79.2	0.4 (0.06)			
tshba	*gh*	52.8	46.7	0.5 (0.03)			
tshba	*pomca*	50.5	48.0	1.5 (0.09)			
sl	*prl*						
sl	*gh*						
sl	*pomca*						
prl	*gh*	81.1	18.5	0.4 (0.06)	81.4	18.2	0.4 (0.05)
prl	*pomca*	80.1	19.7	0.2 (0.03)			
gh	*pomca*						
